# Downregulation of HMGCS2 mediated AECIIs lipid metabolic alteration promotes pulmonary fibrosis by activating fibroblasts

**DOI:** 10.1186/s12931-024-02816-z

**Published:** 2024-04-24

**Authors:** Juntang Yang, Xin Pan, Min Xu, Yingge Li, Chenxi Liang, Lulu Liu, Zhongzheng Li, Lan Wang, Guoying Yu

**Affiliations:** State Key Laboratory Cell Differentiation and Regulation, Henan International Joint Laboratory of Pulmonary Fibrosis, Henan Center for Outstanding Overseas Scientists of Pulmonary Fibrosis, College of Life Science, Henan Normal University, Xinxiang, 453007 Henan China

**Keywords:** Pulmonary fibrosis, AECIIs, Lipid metabolism, HMGCS2

## Abstract

**Background:**

Abnormal lipid metabolism has recently been reported as a crucial signature of idiopathic pulmonary fibrosis (IPF). However, the origin and biological function of the lipid and possible mechanisms of increased lipid content in the pathogenesis of IPF remains undetermined.

**Methods:**

Oil-red staining and immunofluorescence analysis were used to detect lipid accumulation in mouse lung fibrosis frozen sections, Bleomycin-treated human type II alveolar epithelial cells (AECIIs) and lung fibroblast. Untargeted Lipid omics analysis was applied to investigate differential lipid species and identified LysoPC was utilized to treat human lung fibroblasts and mice. Microarray and single-cell RNA expression data sets identified lipid metabolism-related differentially expressed genes. Gain of function experiment was used to study the function of 3-hydroxy-3-methylglutaryl-Coa Synthase 2 (HMGCS2) in regulating AECIIs lipid metabolism. Mice with AECII-HMGCS2 ^high^ were established by intratracheally delivering HBAAV2/6-SFTPC- HMGCS2 adeno-associated virus. Western blot, Co-immunoprecipitation, immunofluorescence, site-directed mutation and flow cytometry were utilized to investigate the mechanisms of HMGCS2-mediated lipid metabolism in AECIIs.

**Results:**

Injured AECIIs were the primary source of accumulated lipids in response to Bleomycin stimulation. LysoPCs released by injured AECIIs could activate lung fibroblasts, thus promoting the progression of pulmonary fibrosis. Mechanistically, HMGCS2 was decreased explicitly in AECIIs and ectopic expression of HMGCS2 in AECIIs using the AAV system significantly alleviated experimental mouse lung fibrosis progression via modulating lipid degradation in AECIIs through promoting CPT1A and CPT2 expression by interacting with PPARα.

**Conclusions:**

These data unveiled a novel etiological mechanism of HMGCS2-mediated AECII lipid metabolism in the genesis and development of pulmonary fibrosis and provided a novel target for clinical intervention.

**Supplementary Information:**

The online version contains supplementary material available at 10.1186/s12931-024-02816-z.

## Background

Pulmonary fibrosis (PF) constitutes the end stage of a broad range of heterogeneous interstitial lung diseases, characterized by the destruction of the pulmonary parenchyma, deposition of extracellular matrix and dramatic changes in the phenotype of both fibroblasts and alveolar epithelial cells [1, 2]. Idiopathic pulmonary fibrosis (IPF), the most common and well-characterized form of pulmonary fibrosis, is a lethal disease with a median survival of 3–5 years upon diagnosis [3, 4]. The most recent data show that from 2004 to 2017, the number of inpatient deaths from IPF in the United States continued to increase [5]. The latest study found that the adjusted incidence of IPF (0.35–1.30 per 10,000 people) and the adjusted prevalence of IPF (0.57–4.51) were both higher in the Asia-pacific region than in the rest of the world [6], despite extensive research efforts and two FDA approved drugs, it remains a significant health burden and an unmet therapeutic need [2]. Currently, there is no cure for PF except for lung transplantation; therefore, revealing the potential pathogenic factors and possible mechanisms would contribute to the prevention and treatment of this deadly disease.

The current paradigm of IPF proposes repeated cycles of injury to lung alveolar epithelial cells, stimulating the recruitment and activation of tissue fibrogenic cells, leading to fibrosis and end-stage organ failure. Recent studies showed that metabolism deregulation was a key feature of IPF [7–9], and clinical studies demonstrated that abnormal lipid was detected in the serum and Bronchoalveolar Lavage Fluid (BALF) in both IPF patients and experimental mice model [10–12]. However, the origin of the lipid remained unknown, and the biological function and possible mechanisms of increased lipid in the process of IPF were still undetermined.

In the current study, we studied the effects and mechanisms of abnormal lipid accumulation in IPF. Our data showed that damaged AECIIs were the primary source of accumulated lipids in response to Bleomycin stimulation. Furthermore, we showed that LysoPCs released by injured AECIIs could activate lung fibroblast in vitro and promote pulmonary fibrosis progression in vivo. We further confirmed that HMGCS2 played a vital role in mitigating lung fibrotic progression via modulating lipid degradation in AECIIs through promoting CPT1A and CPT2 expression by interacting with PPARα.

In summary, this study unveiled a novel etiological mechanism of lung fibrosis and provided a new target for clinical intervention.

## Methods

### IPF patient tissue and serum samples

The diagnosis of IPF was based on ATS/ERS/JRS/ALAT Clinical Practice Guidelines. Tissue samples of IPF were surgical remnants of biopsies from patients undergoing pulmonary transplants at Henna Provincial Chest Hospital. Serum samples of IPF and healthy volunteers were also collected at Henna Provincial Chest Hospital. The study was approved by the Henan Provincial Chest Hospital Medical Research Ethics Committee.

### Cell line and culture

The human alveolar type II-like epithelial cell line A549 [13, 14] and human lung fibroblasts IMR-90 were purchased from the Cell Bank of the Chinese Academy of Science (Shanghai, China). All cells were cultured in the corresponding medium (recommended by the suppliers) supplemented with 10% fetal bovine serum and maintained at 37 °C incubator with 5% CO_2_.

### Hydroxyproline assay

Lung hydroxyproline was analyzed with a hydroxyproline colorimetric assay kit (MAK008, Sigma) following the manufacturer’s instruction, as we previously described [15, 16]. Data are expressed as μg of hydroxyproline/right lung.

### Preparation of cell culture supernatant and lung fibroblast treatment

A549 cells were treated with Belomycin (20 μM) for 48 h, and the supernatant was collected and diluted with lung fibroblast culture medium (1:1). The diluted medium was subsequently subjected to IMR-90 treatment for 24 h. Cells were collected for protein expression analysis.

### Western blot (WB) analysis

Western blot was performed as previously described [16]. After incubation with the secondary antibody, the proteins were detected by chemiluminescence. Primary antibodies used in this study were anti-HMGCS2 (for WB) (1:2000; Abcam ab137043), anti-α-SMA (1:2000; Abcam, ab7817), anti-Collagen1 (1:1000; Cell Signaling Technology, #72026), anti-Fibronectin (1:2000; Cell Signaling Technology, #26836), anti-PPARα (1:1000; Affinity, AF5301), anti-CPT1A (1:1000; Affinity, AF5301), anti-CPT2 (1:1000; Affinity, AF5301), and anti-GAPDH (1:4000; Beyotime, China).

### Immunohistochemical (IHC) analysis

IHC analysis was performed as we previously described [17]. Briefly, mouse lung tissues were embedded in paraffin, sectioned, and rehydrated; these antigens were recovered by heating in relative buffer at 95 °C for 10 min following the antibody instructions (anti-HMGCS2 (1:200; Abcam ab137043)). Endogenous peroxidase was neutralized using the endogenous peroxidase-blocking solution. Afterwards, the lung sections were incubated with a primary antibody at 4 °C overnight. After biotin-labelled secondary antibodies were incubated at 37 °C for 30 min, the lung sections were developed with DAB working solution and then stained with hematoxylin and mounted with a mounting medium.

### Immunofluorescence (IF) analysis

For IF, anti-HMGCS2 (1:200; Mouse monoclonal, sc-393,256, Santa Cruz, USA) and anti-PPARα (1:100; Rabbit Polyclonal, AF5301, Affinity Biosciences) were used, and the protocol was described [15].

### Mouse model

8-week-old female pathogen-free female C57BL/6 mice were purchased from Charles River Experimental Animal Technology Co. Ltd. (China; License Number.

SCXK 2016–0006) and maintained at the appropriate biosafety level at constant temperature and humidity with a 12 h light cycle. Mice lung fibrosis model was established as we previously reported [15]. Briefly, mice were anesthetized with 40% isoflurane and then intratracheally delivered with Bleomycin (Hisun Pharmaceuticals Co. Ltd) 1.5 U/kg diluted in Saline or Saline only (as control) using 22G catheter (GE Healthcare). All experimental animal procedures were approved by the Institutional Animal Care and Use Committee of Henan Normal University, China.

### LysoPC treatment of lung fibroblast and mice

Two commercial available LysoPCs: LysoPC (16:0) HMDB0240262 (CAS Number 66757–27-5, Toronto Research Chemicals, Toronto, Canada) and LysoPC(14:0) HMDB0010379 (CAS Number 20559–16-4, APExBio, Houston, USA) were dissolved in DMSO to make stock solution. For in vitro lung fibroblast treatment, LysoPCs were added to the culture medium with a final concentration of 40 μM and maintained for 48 h. For *the* in vivo mice study, the LysoPC(14:0) was intratracheally delivered, as mentioned above, at a dose of 12.5 mg/kg mouse.

### Isolation of primary AECIIs in mice

Mice AECIIs were isolated using a modified protocol of a previously described method [18]. Briefly, mice lung was perfused with 20 mL wash buffer (DPBS without Ca^2+^ and Mg^2+^ and 0.2 mM EDTA) and instilled with 2 mL digestion buffer (1 mg/mL Elastase and 100 unit/mL DNase I) from the trachea (sealed with 0.5 mL low melted agarose (2% in DPBS)) and digested in a 15 mL tube for 45 min at RT. The lung was then minced by MACS dissociator and filtered successively through 100, 70, 40, 20 μm cell strainer followed by percoll gradient centrifugation (300 g, 20 min). Cells in the middle layer were re-suspended with culture medium and cultured on CD45/32/16 (Biolegend, USA) pre-coated dish to remove immune cells. Using this protocol, a purity exceeding 90% AECIIs was obtained.

### Establishment of HMGCS2 stable cell line

Human alveolar type II-like epithelial cells A549 that stably expressed HMGCS2 were established according to our previously described [19]. Briefly, The PCDNA3.1 -EGFP-T2A -HMGCS2 vector was transfected using Lipo3000 transfection reagent (Invitrogen, USA). The stably transfected cells were screened with 800 μg/mL G418 (Calbiochem, La Jolla, CA, USA). A single clone was obtained by the cylinder method, as we previously described [19]. Several positive clones were confirmed by WB and then mixed for consequent experiments.

### Co-immunoprecipitation

Total extracts of A549 cells with or without ectopic expression of HMGCS2 were lysed with co-IP lysis buffer (Beyotime, China). The cell lysate was incubated with primary antibody rabbit anti-HMGCS2 (1:50, Cell Signaling Technology, #20940) at 4 °C overnight with rotation. On the second day, incubate the antibody lysate mixture with Protein G Agarose (Fast Flow, for IP, P2053, Beyotime, China) at 4 °C for 4 h with rotation and wash with co-IP lysis buffer for 3 times. Subsequent WB analyses were performed as described above. To avoid the effects of denatured and reduced rabbit IgG heavy chain, the mouse anti-rabbit IgG conformation-specific HRP conjugate secondary antibody (1:2000, Cell Signaling Technology, #L27A9) was used for WB detection of target band after immunoprecipitation.

### Generation of HMGCS2 overexpression system in AECIIs

The adeno-associated virus (AAV) system was used to generate mice with HMGCS2 specifically expressed in AECIIs. Briefly, HMGCS2 cDNA was cloned into the pHBAAV-SPC-T2A-ZsGreen vector with AECIIs specific promoter SPC**.** Subsequently, the pHBAAV-SPC- HMGCS2-T2A-ZsGreen vector was co-transfected into 293 T cells with pAAV2 Rep/AAV6 Cap and pHelper in a 1:1:2 ratios using Lipofiter™ (Hanbio Biotechnology, HB-TRCF-1000, China). After 72 h of transfection, the cells were lysed, and the crude viral lysates were purified using an AAV purification kit (Biomiga, V1469–01, USA) according to the protocol. Virus stock was stored in − 80 °C before use. To generate mice with HMGCS2 specifically expressed in AECIIs, mice were anesthetized with 40% isoflurane and then intratracheally delivered with HBAAV2/6-SPC-HMGCS2-T2A-ZsGreen and HBAAV2/6-SFTPC-T2A-ZsGreen virus (5 × 10^10^ vg/mouse, 50 μL) using 22G catheter (GE Healthcare). These mice were maintained for 3 weeks, and the infection efficiency was confirmed by IF and WB before being subjected to the establishment of a lung fibrosis model. All animal experimental procedures were approved by the Institutional Animal Care and Use Committee of Henan Normal University.

### Untargeted lipid omics analysis

Untargeted Lipid omics analysis was conducted in cooperation with Major Biotechnology (Shanghai, China). Briefly, 200 μL of liquid samples (cell culture supernatant and IPF serum) were accurately pipetted into a 1.5 mL centrifuge tube and 80 μL methanol and then 400 μL methyl tert-butyl ether followed by sonication for 30 min (4 °C, 40 KHz), and placed at − 20 °C for 30 min.

The samples were centrifuged for 15 min (13,000 g, 4 °C), and 350 μL of the supernatant was transferred into an EP tube and dried with nitrogen. 100 μL of extract buffer (isopropanol: acetonitrile = 1:1) was added to the nitrogen-dried sample and followed by low-temperature ultrasonic extraction for 5 min (4 °C, 40KHz).

After centrifugation (13,000 g, 4 °C), the supernatant was pipetted into an injection vial for ultra-high-performance liquid chromatography-mass spectrometry analysis using a Vanquish Horizon UHPLC system (Thermo Scientific, USA) coupled to a Q Exactive HF-X mass spectrometer (Thermo Scientific, USA). The raw data was imported to Lipidsearch (Thermo Fisher, USA) for baseline filtering, peak identification, integration, retention time correction, peak alignment and finally, a data matrix with retention time, mass-to-charge ratio and peak intensity was obtained. The software was then used to identify the characteristic peak by matching the MS and MS/MS mass spectrometry information with the metabolic database. The MS mass error was set to less than 10 ppm, and the metabolites were identified according to the secondary mass spectrometry matching score.

### Oil red staining

Saline or Bleomycin-treated cells were washed with PBS and fixed with 4% paraformaldehyde for 20 min at RT. Then, the cells were washed with sterile water and treated with 60% isopropanol for 5 minutes at RT. Finally, cells were stained with an oil-red working solution for 7 min and washed with sterile water, and nuclei were stained with hematoxylin. 3 random fields were chosen under a microscope for further analysis. Fresh frozen mice lung slides were fixed with 4% paraformaldehyde and washed with sterile water. Then, the slides were pretreated with propylene glycol followed by oil red O (O0625, Sigma-Aldrich, Saint Louis, MO, USA) staining for 10 minutes. After that, the slides were washed with 85% Propylene glycol, and nuclei were stained with hematoxylin. For lung tissues we used frozen section (10 um thick) rather than traditional paraffin section and all slide were neither dehydrated nor treated with xylene after nuclei staining. Finally, slides were mounted with glycerin jelly, and 3 random fields were chosen under a microscope for further analysis.

### Nile red staining and FACS analysis

Bleomycin-treated Vector-A549 and HMGCS2-A549 cells were collected and washed with PBS and stained with Nile red working solution (1 μg/ml in methanol) at 37 °C for 15 min. Then, the cells were suspended in PBS and analyzed by FACS (λex 530 nm; λem 635 nm). Data were further analyzed using FlowJo, and results were presented as the portion of Nile red-positive cells.

### Site-directed point mutation

To generate HMGCS2 L393V point mutation, the wild type h-HMGCS2 was mutated using the QuickMutation™Plus kit (D0208M, Beyotime, China) with slight modification according to our previous protocol [20]. Briefly, mutagenic primers were used to amplify the PCDNA3.1-T2A-EGFP-HMGCS2 plasmid. The un-mutated plasmid template was eliminated by DpnI digestion, and then the DpnI-treated DNA was transformed into DH5α ultracompetent cells. The mutations were validated by sequencing. The primers used in the site-directed mutagenesis were listed in Supplemental Table S1.

### Analysis of public datasets

For microarray analysis, our previous microarray data (GSE47460) was reanalyzed using R and for the GEO. We use the R package GEO query to download the data. Get clinical data using the pData function. Subsequently, we used the R package Annoprobe to convert the probe names of the two platforms (GPL6480, GPL14550) into gene names. Use the merge function to merge data from different platforms. PCA method was used to reduce dimension to observe whether batch effect existed.

### Single cells data analysis

Raw data of Single-cell sequencing of lung epithelial cells from 3 IPF patient samples (Fibrotic region) and 3 donor control samples were downloaded from GEO (GSE132915) [21] and followed by the route single-cell pipeline as we previously described [22].

### Statistical analysis

Data were processed with Prism version 5.0 (GraphPad, San Diego, CA, USA) and are expressed as mean ± SD. The significance of the differences between two groups was analyzed using a two-tailed Student’s t-test and One-way analysis of variance was used for multi-group comparisons followed by Tukey’s multiple comparison. *p* < 0.05 was considered statistically significant.

## Results

### Lipid accumulation is obvious in AECIIs in experimental lung fibrosis

**Fig. 1:**
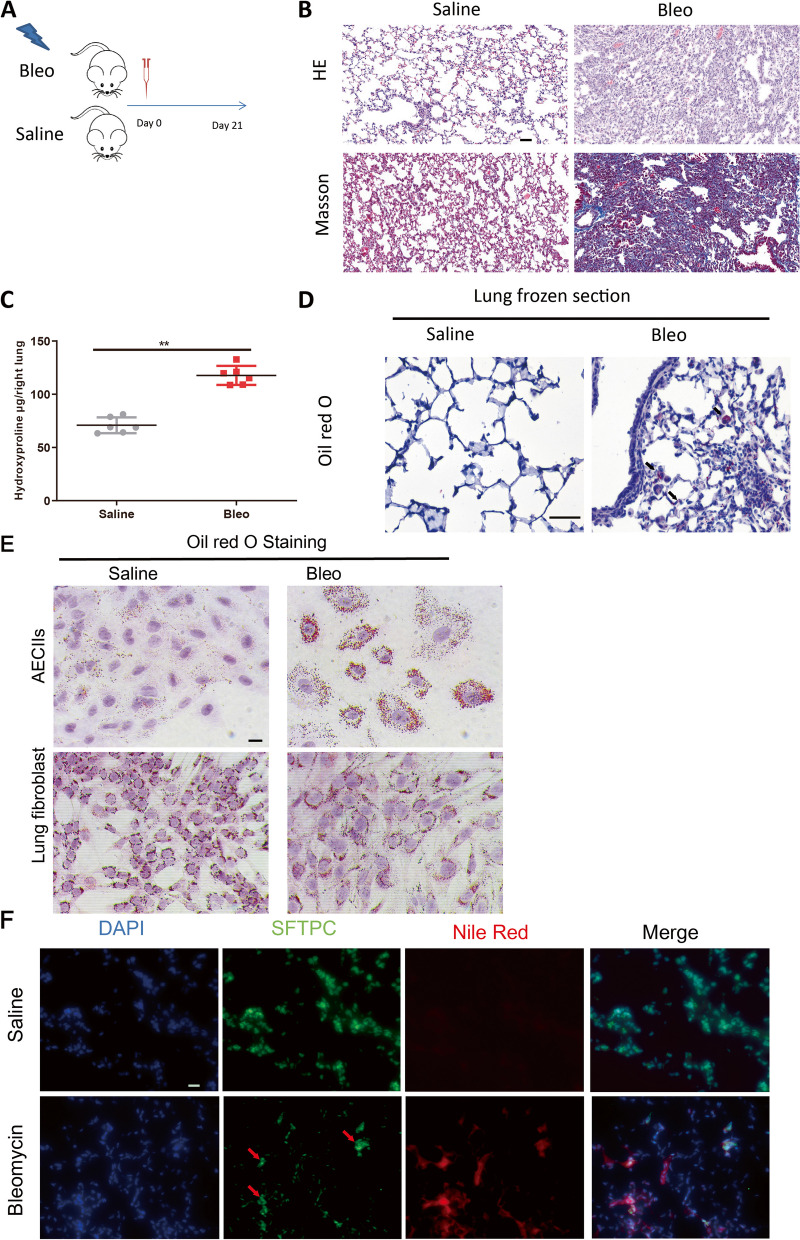
Lipid accumulation was obviously found in AECIIs in mice lung fibrosis. **A** The scheme establishment of mouse lung fibrosis model. **B**, **C**, HE staining and Hydroxyproline content confirmed the mice lung fibrosis model, scale bar = 50 μm. **D**, Oil red staining showed significant lipid accumulation in mice lung fibrosis section arrow indicate the positive cells, scale bar = 50 μm. **E**, In vitro oil red staining showed that upon Bleomycin injury, dramatic lipid accumulation was only found in lung alveolar epithelial cells rather than lung fibroblast, scale bar = 20 μm. F, Nile red and SFTPC co-staining indicated the lipid was observed specifically in AECIIs, arrow indicate the SFTPC positive cells, scale bar = 20 μm. Data are expressed as mean ± SD, *: *p* < 0.05; **: *p* < 0.01

**Fig. 2:**
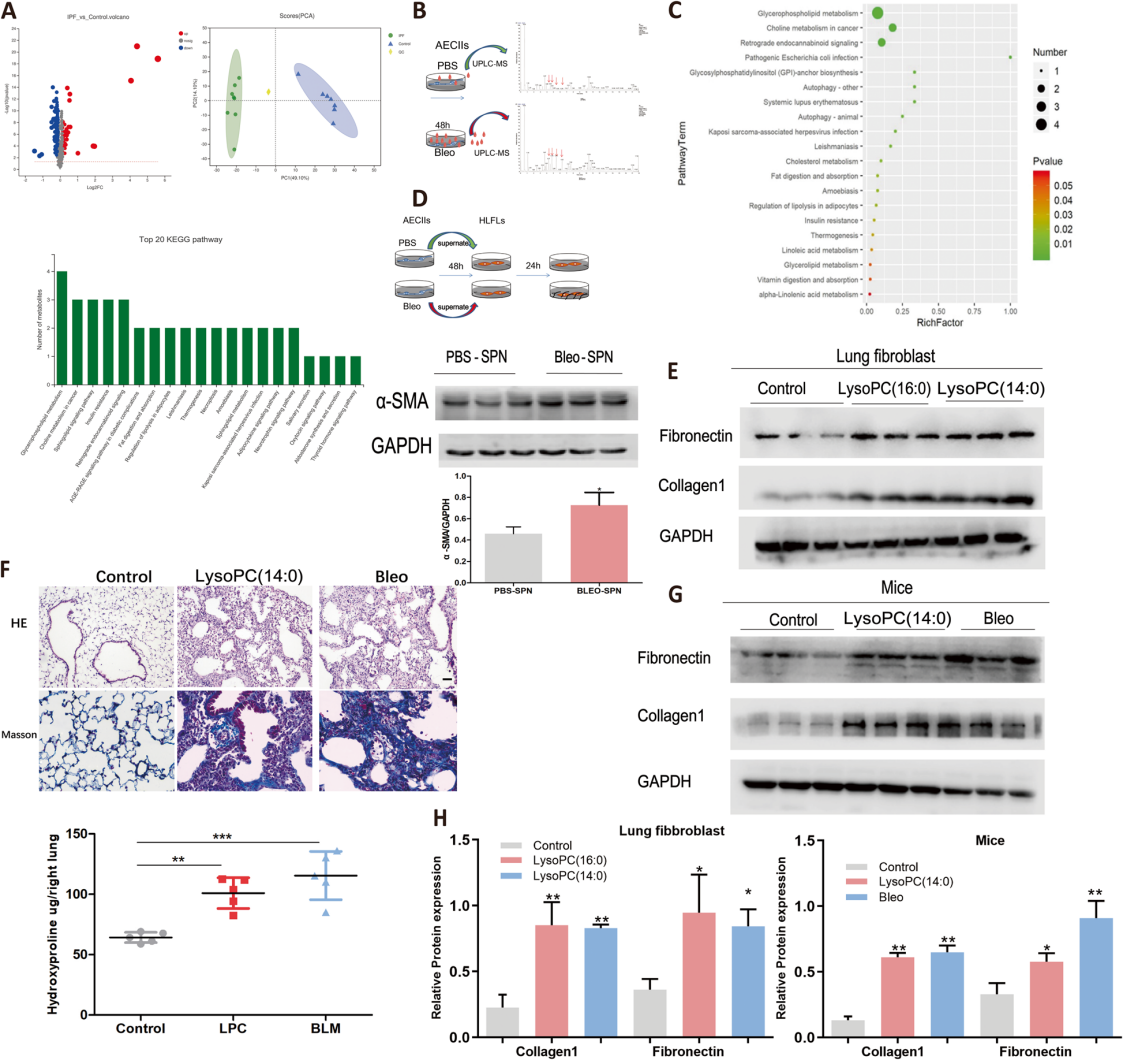
LysoPC activated lung fibroblast and promoted mice lung fibrosis. **A** UPLC-MS analyzed the lipid signature of serum of IPF patient, principal components analysis of overall serum profiles of lipid molecules in IPF patients (*n* = 8) and healthy control (*n* = 7) (upper); KEGG pathway analysis showed that top alter lipid metabolic pathway was enriched in glycerophospholipid and choline metabolism (lower). **B**, UPLC-MS analyzed the lipid signature of Bleomycin treated A549 supernatant. **C**, GO analysis showed the top alter lipid metabolic pathway was enriched in glycerophospholipid and choline metabolism. **D**, Bleomycin treated A549 supernatant significantly activated lung fibroblast. **E**, LysoPC identified from UPLC-MS could activate lung fibroblast in vitro. **F**,**G**, LysoPC (14:0) promoted experimental mice lung fibrosis which was comparable to the effects of Bleomycin as showed by histological analysis, scale bar = 20 μm, hydroxyproline content and western blot detection of fibrotic markers. **H**, Relative protein expression analysis of fibrotic markers of lung fibroblast and mice upon treatment of LysoPC. Data are expressed as mean ± SD, *: *p* < 0.05; **: *p* < 0.01

To determine the status of lipids metabolism in fibrotic lungs, we first established a pulmonary fibrosis mice model (Fig. 1A-C), and oil red staining showed that accumulated lipid was obviously found in fibrosis lung sections (Fig. 1D). To unveil the specific type of cell that accumulated lipid, in vitro experiments were first used to examine the lipid contents upon Bleomycin stimulation. Alveolar epithelial cells and interstitial fibroblasts are composed of a large number of alveolar structural cells. Therefore, a widely used human alveolar type II-like epithelial cell line A549 (A549 is usually used as a replacement for primary AECIIs, which are difficult to obtain and maintain in culture ex vivo [13, 14]) and lung fibroblast IMR-90 were subjected to Bleomycin treatment for 48 h, significant lipid accumulation was observed only in AECIIs cells but not in lung fibroblast (Fig. 1E). To further validate this observation in vivo, SFTPC (AECII specific marker) and Nile red (a selective fluorescent stain for intracellular lipid droplets) [23] co-staining of frozen lung section demonstrated that lipid droplets predominantly existed in AECIIs (Fig. 1F). To further confirm lipid metabolism was dysregulated in IPF patients, serum samples of 8 IPF and 7 healthy controls were subjected to lipidomics analysis. A clear separation was observed between IPF and control group using principal components analysis, and Kyoto Encyclopedia of Genes and Genomes (KEEG) analysis showed that the top enriched pathways were glycerophospholipid and choline metabolism (Fig. 2A). Taken together, these data indicated that abnormal lipid metabolism mainly occurred in AECIIs in the process of lung fibrosis.

### LysoPC released by AECIIs promotes lung fibrosis progression

Next, we sought to explore the possible biological consequences of lipid accumulation in AECIIs. Our data, together with previous studies, showed abnormal lipid signatures in the serum of IPF patients and mice lung fibrosis model [12, 24]. Thus, we speculated the lipid could be released by the Bleomycin-injured AECIIs and subsequently participate in the process of lung fibrosis. To test this hypothesis, a Bleomycin-treated A549 cell culture medium was collected to incubate lung fibroblasts (Fig. 2B,D). The supernatant could significantly activate lung fibroblast as evidenced by elevated α-SMA expression (Fig. 2D). The supernatant was subjected to UPLC-MS and KEGG analysis showed the top altered lipid metabolic pathway was glycerophospholipid and choline metabolism (Fig. 2C), which was consistent with lipid omics data of our IPF serum sample (Fig. 2A). Based on the KEGG analysis, we hypothesized that LysoPC, which was derived from glycerophospholipid with the substitution of choline [10, 25], might be the vital lipid involved in the process of fibrotic progression. To further unveil what specific type of LysoPC species could activate lung fibroblast, we selected the top-ranked LysoPCs with compound ID which were commercialized available (Table S2) and confirmed that LysoPC(14:0) HMDB0010379 and LysoPC (16:0) HMDB0240262 could activated lung fibroblast in vitro (Fig. 2E) and facilitated experimental lung fibrosis in vivo (Fig. 2F, G). These results suggested that LysoPCs derived from the injured AECIIs could contribute to the fibrotic phenotype of lung fibroblast and promote lung fibrosis progression in mice.

### HMGCS2 regulates AECIIs lipid accumulation upon bleomycin injury

**Fig. 3:**
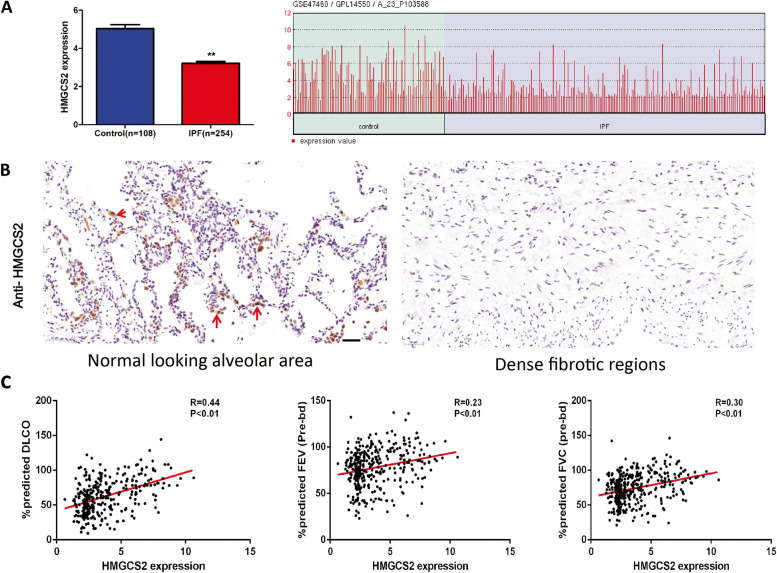
HMGCS2 was down regulated in IPF patients and associated with lung function. **A**, HMGCS2 was significantly down regulated in IPF patients (*N* = 254) compared with healthy individuals (*N* = 108) (GES47460). **B**, The expression pattern of HMGCS2 was further confirmed in IPF lung sections (Normal looking alveolar area VS dense fibrotic region), scale bar = 20 μm. **C**, HMGCS2 expression was positively correlated with lung function in IPF cohort (GES47460) (DLCO, R = 0.44, *P* < 0.01), forced expiratory volume (FEV, R = 0.23, P < 0.01), and forced vital capacity (FVC, R = 0.30, P < 0.01), Data are expressed as mean ± SD, *: *p* < 0.05; **: *p* < 0.01

**Fig. 4:**
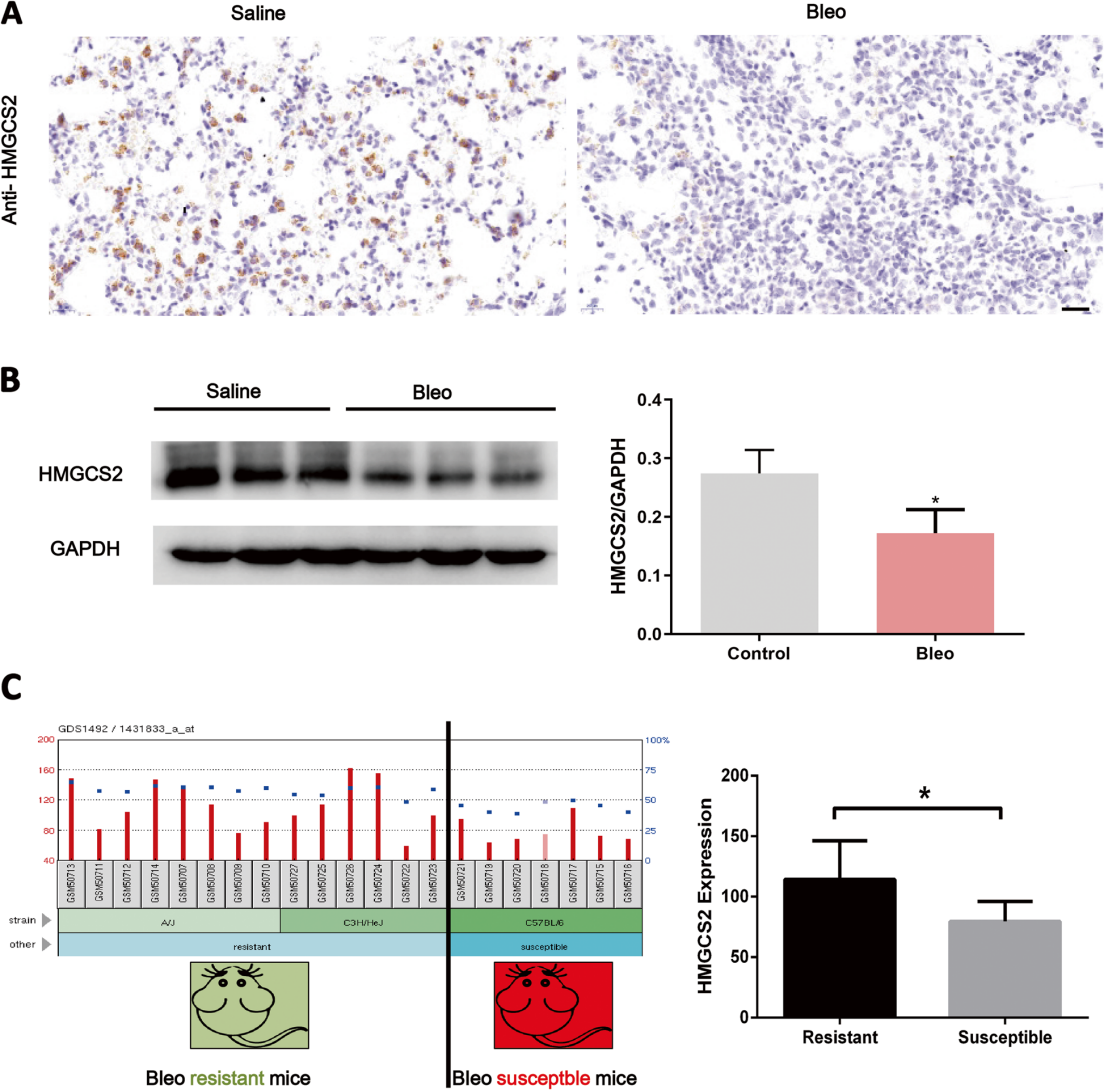
HMGCS2 was down regulated in Bleomycin induced mice lung fibrosis model and associated with Bleomycin resistance. **A**-**B**, HMGCS2 was significantly down-regulated in mice lung fibrosis model, scale bar = 20 μm. **C**, HMGCS2 expression was lower in Bleomycin susceptible mice strain C57BL6 (GDS1492). Data are expressed as mean ± SD, *: *p* < 0.05; **: *p* < 0.01

**Fig. 5:**
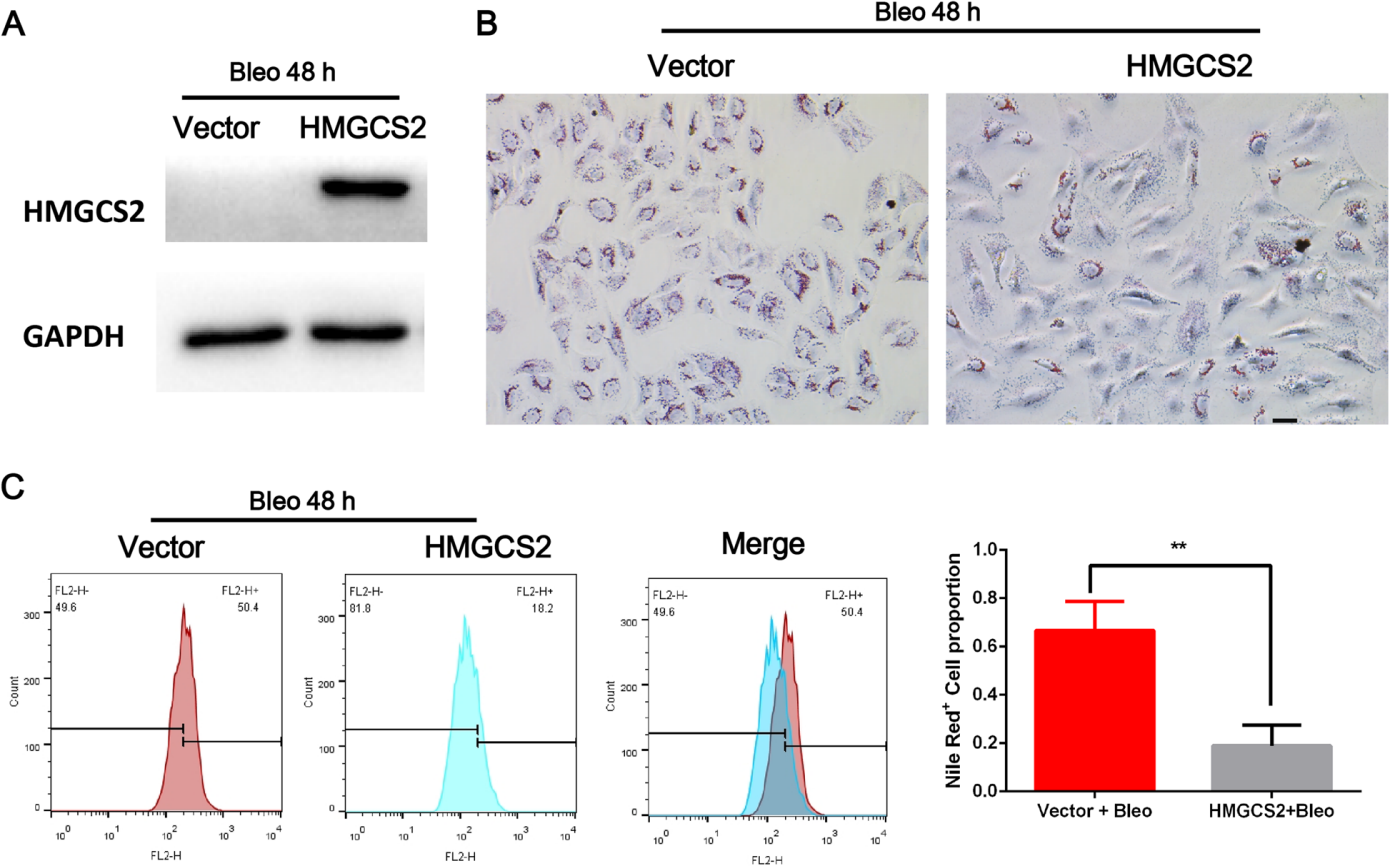
Ectopic expression of HMGCS2 inhibited Bleomycin induced lipid accumulation in A549 cells. **A**, The stable expression of HMGCS2 in A549 cells was confirmed by western blot. **B**, Oil red staining indicated that ectopic expression of HMGCS2 inhibited Bleomycin induce lipid accumulation in A549 cells, scale bar = 20 μm. **C**, Nile staining followed by FACS analysis showed that ectopic expression of HMGCS2 inhibited Bleomycin induce lipid accumulation in A549 cells. Data are expressed as mean ± SD, *: *p* < 0.05; **: *p* < 0.01

To elucidate the possible mechanisms of abnormal lipid accumulation in AECIIs in the process of lung fibrosis, we reanalyzed IPF microarray data (GES47460) with clinical information used in our previous study to find differentially expressed lipid metabolism-related genes [15] and further validated using four external IPF data sets (Fig. S1). A mitochondrial-located lipid metabolic gene 3-hydroxy-3-methylglutaryl-Coa Synthase 2 (HMGCS2) was significantly decreased in all the data sets (Fig. 3A and Fig. S1) and the protein level of HMGCS2 was further confirmed using IHC in IPF lung sections (Fig. 3B) and mice lung fibrosis model (Fig. 4A, B). Clinically, the expression of HMGCS2 was positively correlated with lung function in the IPF cohort (GES47460), including diffusing capacity for carbon monoxide (DLCO, R = 0.44, *P* < 0.01), forced expiratory volume (FEV, R = 0.23, P < 0.01), and forced vital capacity (FVC, R = 0.30, P < 0.01) (Fig. 3C) and negatively correlated with age (risk factor of IPF) (Fig. S2) suggesting its vital role in the pathogenesis of IPF. Interestingly, by analyzing the GEO data set (GDS1492), we found that the C57BL/6 mice strain (a widely used mice strain for establishing Bleomycin lung fibrosis), which was more susceptible to Bleomycin injury, showed lower HMGCS2 expression (Fig. 4C). These data strongly suggested that HMGCS2 played a vital role in the pathological development of lung fibrosis. By analyzing a public single-cell data analyzing platform (http://ipfcellatlas.com/) and another independent GEO lung epithelial single-cell RNAseq data set (GSE132915), we found that HMGCS2 was specifically downregulated in AECIIs in IPF (Fig. S3A-C) and isolated mice AECIIs in Bleomycin group indeed showed significant down-regulation of HMGCS2 (Fig. S3D). The results indicated that a specific deficiency of HMGCS2 in AECIIs was vital for lung fibrosis progression. Therefore, to investigate whether HMGCS2 could reverse Bleomycin-induced lipid accumulation in AECIIs, we developed stable expression of HMGCS2 in A549 cells (Fig. 5A). The A549-HMGCS2 cells were subjected to Bleomycin injury, and lipid content was evaluated by FACS and oil red staining (Fig. 5B, C). These results demonstrated that HMGCS2 re-expression could significantly reduce lipid content after Bleomycin injury in A549 cells. Taken together, the above results strongly suggest that deficiency of HMGCS2 in AECIIs is crucial for the pathological origin or progression of lung fibrosis by compromising lipid metabolism.

### Overexpression of HMGCS2 in mice AECIIs inhibits lipid accumulation and lung fibrosis in vivo

**Fig. 6:**
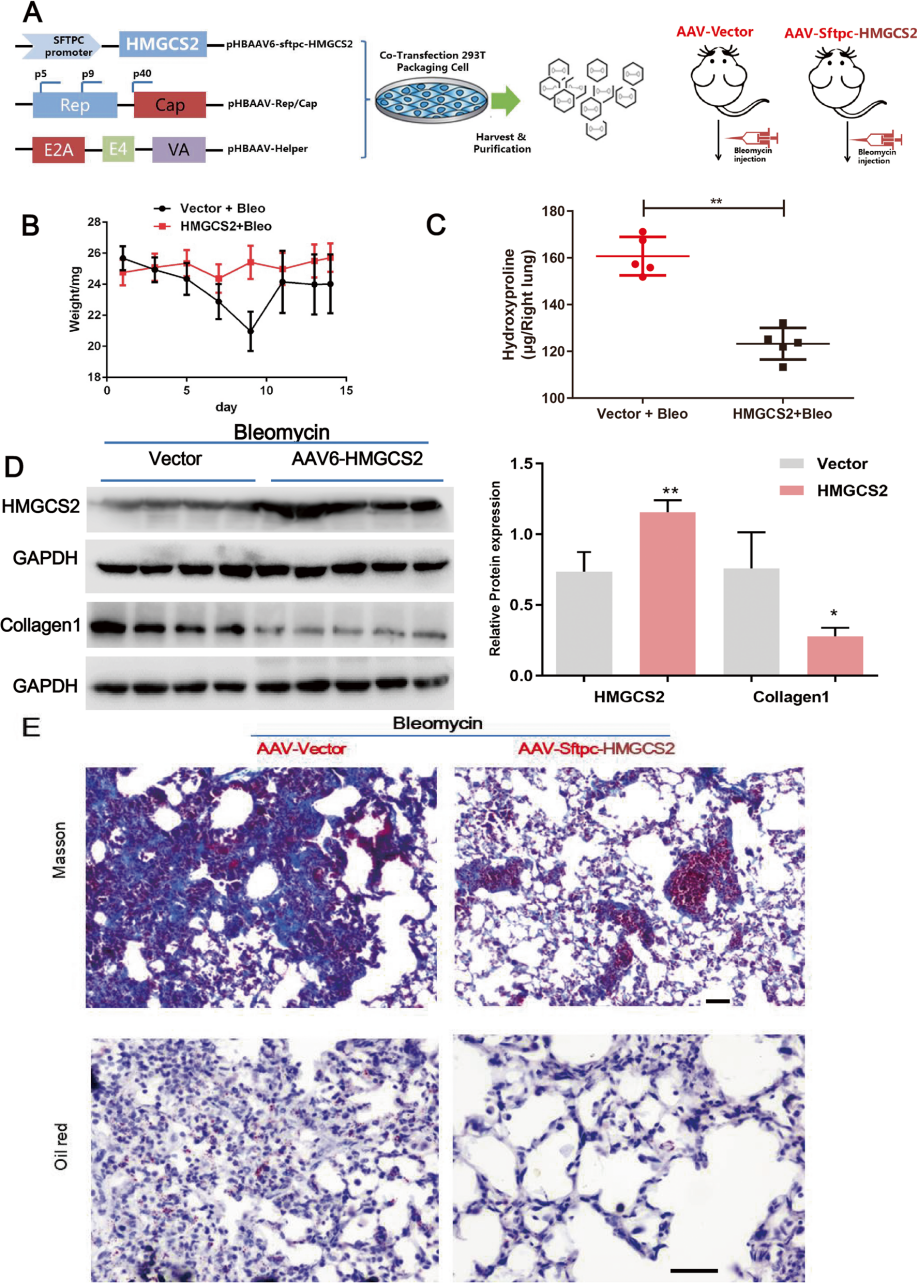
Ectopic expression of HMGCS2 in mice AECIIs mitigated Bleomycin induced lung fibrosis and lipid accumulation. **A**, Scheme of the establishment of mice with AECIIs overexpressing HMGCS2, wild-type C57BL/6 J mice were administered HBAAV2/6-SPC-T2A-ZsGreen and HBAAV2/6-SPC-mHMGCS2-T2A-ZsGreen virus and Bleomycin was intratracheally delivered 3 weeks later to establish lung fibrosis. **B**,**C**, Body weight curve and hydroxproline content after Bleomycin treatment. **D**, HMGCS2 expression and Collagen1 content were determined by western blot. **E**, Masson’s Trichrome and Oil red staining of lung sections to visualize collagen deposition and lipid accumulation, scale bar = 50 μm. Data are expressed as mean ± SD, *: *p* < 0.05; **: *p* < 0.01

To further validate the anti-fibrotic role of HMGCS2, we established a mice model with HMGCS2 specifically overexpressed in mice AECIIs by intratracheal instillation of AAV2/6-SFTPC or AAV2/6-SFTPC-HMGCS2 (HMGCS2 expression was regulated under the control of AECIIs specific promoter SFTPC) (Fig. 6A). The infection efficiency was confirmed by IF and WB before and after exposure to Bleomycin (Fig. 6D and S4). HMGCS2 overexpression in AECIIs could significantly blunt the development of lung fibrosis as evidenced by reduced tissue hydroxyproline content, Collagen1 and collagen deposition (Fig. 6C, D, E upper panel) while the body weight showed no difference at the endpoint (Fig. 6B). Furthermore, oil-red staining showed that HMGCS2 overexpression decreased lipid accumulation upon Bleomycin injury, which further confirmed that HMGCS2 ameliorated lung fibrosis by facilitating lipid metabolism in AECIIs (Fig. 6E lower panel). Taken together, these data indicated that HMGCS2 could mitigate Bleomycin-induced lung fibrosis by decreasing lipid accumulation in AECIIs.

### HMGCS2 interacted with PPARα to facilitate AECIIs lipid metabolism by stimulating CPT1A and CPT2 expression

**Fig. 7:**
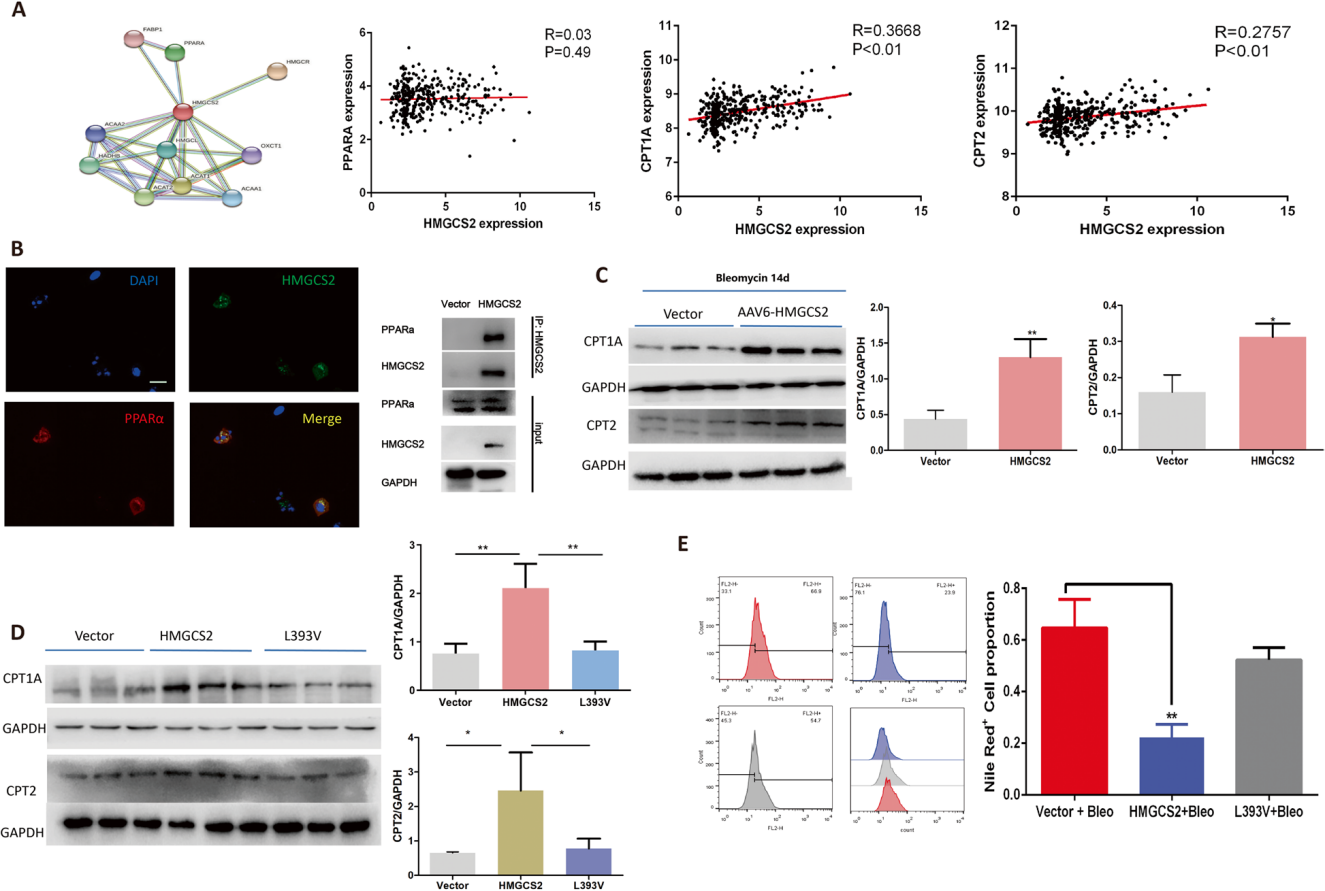
HMGCS2 interacted with PPARα to facilitate lipid degradation metabolism in AECIIs. **A**, HMGCS2 was predicted to interacted with PPARα and the expression of HMGCS2 was positively associated with PPARα downstream genes CTP1A (R = 0.3668, P < 0.01) and CPT2 (R = 0.2757, P < 0.01) but not correlated to the expression of PPARα (R = 0.03, *P* = 0.49) in IPF cohort (GSE47460). **B**, The interaction between HMGCS2 and PPARα was further confirmed by IF co-localization and Co-ip, scale bar = 20 μm. **C**, CPT1A and CPT2 expression were recovered by adenoviral mediated expression of HMGCS2 in mice AECIIs in Bleomycin lung fibrosis model. **D**, L393V mutation abolished the interaction of HMGCS2 with PPARα and subsequently failed to recover CPT1A and CPT2 expression in A549 cells upon Bleomycin injury. **E**, L393V mutation HMGCS2 failed to inhibit the lipid accumulation in A549 cells upon Bleomycin treatment. Data are expressed as mean ± SD, *: *p* < 0.05; **: *p* < 0.01

To further explore the mechanism by which HMGCS2 facilitates lipid metabolism in AECIIs. We reanalyzed IPF microarray data and found that HMGCS2 expression was positively correlated with CPT1A and CPT2 but not with PPARα, while potential interaction between HMGCS2 and PPARα was observed using STRING analysis (Fig. 7A). CPT1A and CPT2 were well-known target genes controlled by PPARα [26–28], and palmitoylation of HMGCS2 enhances its interaction with PPARα and expression of target genes in liver cells [29]. A recent study showed that CPT1A and CPT2 were crucial for the remodeling of glycerol phospholipid [27]. Therefore, we assumed that HMGCS2 might act as a transcriptional coactivator of PPARα in regulating the expression of PPARα target genes such as CPT1A and CPT2. The immunoflurence and Co-ip assay were then performed and showed that HMGCS2 could interact with PPARα in A549 cells (Fig. 7B). Furthermore, HMGCS2 could increase the expression of CPT1A and CPT2 in lung fibrosis models in mice (Fig. 7C). HMGCS2 contained a conserved LASLL motif that was necessary for its binding to PPARα [29, 30]. To confirm the interaction between PPARα was crucial for increasing CPT1A and CPT2 expression and regulating AECIIs lipid metabolism, a point mutation replacement of Leu393 with valine (L393V) [30] in the LASLL motif of HMGCS2 was generated to further confirm the interaction of HMGCS2 with PPARα and the data showed that L393V mutation compromised the effects of HMGCS2 on promoting the expression of CPT1A and CPT2 (Fig. 7D) thereby by abolished lipid metabolism of AECIIs in response to Bleomycin injury (Fig. 7E). In conclusion, these data suggested that HMGCS2 facilitated lipid metabolism of AECIIs through interacting with PPARα, which promoted the expression of CPT1A and CPT2 in mice lung fibrosis models.

## Discussion

Recent studies showed that metabolism deregulation was a key feature of IPF [7–9], and clinical studies demonstrated that abnormal lipids were detected in the serum and BALF in both IPF patients and experimental mice [10–12]. However, which type of cells contributed to the dysregulated lipid metabolism phenotype remained unknown, and the subsequent biological function and possible mechanisms of altered lipid metabolism in the process of IPF were still undetermined.

To address the first question, oil red staining was used to examine lipid content in mice fibrotic lung sections. Compared with lung fibroblast, lung alveolar epithelial cells showed enlarged cell morphology and obvious lipid accumulation in response to Bleomycin injury. Further, SFTPC and Nile red co-staining results showed that lipids were mainly accumulated in AECIIs in lung fibrosis. The AECIIs are rich in lipid synthesis and utilization since lipid synthesis is crucial for the normal function of AECIIs since its main function is to secret surfactant to release alveolar tension and pulmonary surfactant synthesized by AECIIs is a lipoprotein complex, with 90% of its mass being lipid and the remaining 10% proteins that are in many cases specific of the alveolar compartment [31]. A recent study showed that the surviving AECII cells regenerate alveolar epithelium after Bleomycin-induced lung injury by repressing fatty acid synthesis [32], which indicated that increased lipid content might not favor the resolution of Bleomycin-induced lung fibrosis. Taken together, these data indicated that abnormal lipid metabolism predominantly occurring in AECIIs might contribute to the process of lung fibrosis.

To explore the biological effects of abnormal lipid metabolism in AECIIs, we hypothesized that lipid could serve as a paracrine signal molecule to affect surrounding fibroblast since lipid was observed in the serum of IPF and mice lung fibrosis [10–12]. Bleomycin-treated A549 cell culture medium was collected to incubate lung fibroblast. The supernatant could significantly activate lung fibroblast as evidenced by elevated α-SMA expression on protein levels. A recent study showed that A549 cells injured with 20 mU Bleomycin could induce senescence [14] while senescent cells could release (senescence-associated secretory phenotype) SASP such as IL-1α, IL-6, IL-8, and matrix metalloproteinase 9 (MMP9) which could act as an extracellular signal molecule to promote fibrotic progression [14, 33]. To further confirm that lipids, in addition to SASP, could also activate lung fibroblasts, we utilized untargeted lipid omics to analyze the lipid species altered in the supernatant.

As expected, the content of many types of lipids, such as PC, LysoPC, PE, PS, PG, sphingolipids, Cer, and SM, was altered. KEGG database analysis of IPF serum and supernatant of Bleomycin treated A549 showed consistently that the top altered lipid metabolic pathways were glycerophospholipid and choline metabolism pathways. Therefore, our target lipid species narrowed down to PC and LysoPC [10, 25]. PC is the most abundant phospholipid in mammalian cells and organelles [10]. As for the lung, it is the major component of surfactant, which could help release the extra tension of the alveolar [31]. However, LysoPCs have recently been identified as a potential biomarker for IPF patients [34], and LPA, which is derived from LysoPC, has been linked to the development of organ fibrosis, such as in the lung kidney [35]. Based on this evidence, we finally focus on LysoPCs. By using commercialized available LysoPC, we found that LysoPC (14:0) HMDB0010379 and LysoPC (16:0) HMDB0240262 could activate fibroblast and facilitate proliferation in vitro and in vivo. These results suggested that LysoPCs derived from the injured AECIIs contribute to the fibrotic phenotype of lung fibroblast. Clinically, trials on small molecules that target Lysophosphatidic acid (LPA) signaling have been widely carried out [36–39]; however, adverse effects such as liver toxicity have emerged [40]. Thus, our results provide a novel target for anti-lung fibrotic drug development.

To elucidate the possible mechanisms by which abnormal lipids accumulated in AECIIs, we analyzed the IPF microarray data previously used in our study and found a mitochondrial lipid metabolism-associated gene HMGCS2 is significantly downregulated in IPF patients. To our knowledge, there is no research regarding the function of HMGCS2 in IPF, except a recent study that identified HMGCS2 as a potential bronchoalveolar lavage fluid biomarker [41]. The expression of HMGCS2 was positively correlated with DLCO, FEV, and FVC, suggesting its vital role in the pathogenesis of IPF. The decreased expression pattern of HMGCS2 was further confirmed in the Bleomycin-induced mice lung fibrosis model. Interestingly, by analyzing the GEO data set, we found that the C57BL/6 mice strain that was more susceptible to Bleomycin-induced pulmonary fibrosis showed lower HMGCS2 expression. These data strongly suggested that HMGCS2 played a vital role in the pathological development of IPF. Single-cell data showed that HMGCS2 was specifically downregulated in AECIIs of IPF patients, and isolated mice AECIIs in the Bleomycin group also exhibited significant downregulation of HMGCS2. The A549-HMGCS2 stable cells were subjected to Bleomycin injury, and lipid content was evaluated. These results showed that HMGCS2 expression could significantly reduce lipid content after Bleomycin injury in A549 cells. Taken together, the above results strongly suggest that deficiency of HMGCS2 in AECIIs is crucial for the pathological origin or progression of IPF by compromising lipid metabolism. We reanalyzed IPF microarray data and found that HMGCS2 expression was positively correlated with CPT1A and CPT2 but not with PPARα. CPT1A and CPT2 were well-known target genes controlled by PPARα [26–28, 42], and palmitoylation of HMGCS2 enhances its interaction with PPARα and expression of target genes in liver cells [29]. A recent study demonstrated that reduced PPARα in AECIIs is associated with senescence and promotes lung fibrosis [43]. Therefore, we assumed that HMGCS2 might act as a transcriptional coactivator of PPARα in regulating the expression of PPARα target genes such as CPT1A and CPT2 in lung fibrosis. The immunoflurence and Co-ip assay were then performed and showed that HMGCS2 could interact with PPARα in A549 cells. Furthermore, HMGCS2 could increase the expression of CPT1A and CPT2 in mice with lung fibrosis. HMGCS2 contained a conserved LASLL motif that was necessary, and sometimes sufficient, for binding to PPARα [29, 30]. A point mutation replacement of Leu393 with valine (L393V) in the LASLL motif of HMGCS2 was generated [30] to further confirm the notion that the interaction with HMGCS2 was vital for PPARα to activate CPT1A and CPT2. Our data showed that the L393V mutation compromised the effects of HMGCS2 on promoting the expression of CPT1A and CPT2, thereby interfering with the lipid metabolism of AECIIs in response to Bleomycin injury. A recent study showed that CPT1A could alleviate kidney fibrosis by restoring mitochondrial homeostasis [44]. In conclusion, these data suggested that HMGCS2 facilitated lipid metabolic of AECIIs through interacting with PPARα to promote the expression of CPT1A and CPT2 in mice lung fibrosis models. A recent study showed that CPT1A and CPT2 were crucial for the glycerol phospholipid remodeling [27], and cigarette smoking-induced mtROS inhibited the expression of PPARα and CPT1A in lung fibrosis [42], which are in line with previous studies demonstrating that PPARα activators exerted anti-fibrotic effects in the lung [45] and another organ such as liver and kidney [42].

The latest study found abnormal mitochondrial function in the liver of HMGCS2^−/−^ newborn mice (by inhibiting over-acetylation of mitochondrial protein) [46]. However, HMGCS2 in popocytes (renal cystic epithelial cells) was associated with mitochondrial damage induced by high fructose intake in mice [47], therefore, the effects of HMGCS2 on AECII mitochondria should be explored in the future since the mitochondria damage of AECIIs is considered involved in the initiation of lung fibrosis.

## Conclusions

**Fig. 8:**
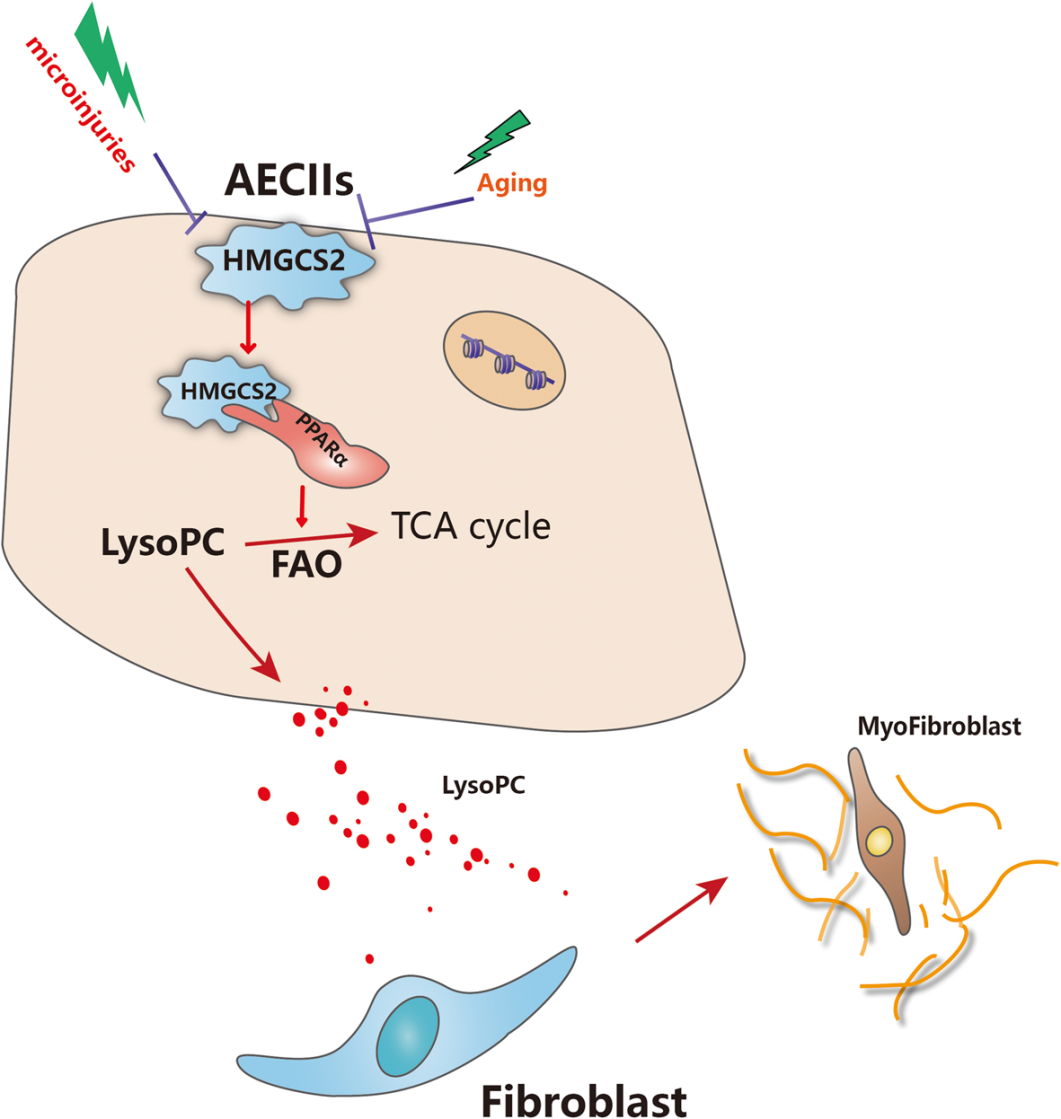
HMGCS2 alleviates lung fibrosis by regulating AECIIs lipid metabolism. Decreased expression of HMGCS2 in AECIIs leads to lipid accumulation. Lipid such as LysoPC determined in our study released by injured AECIIs could activate surrounding lung fibroblast therefore facilitating fibrotic progression

Our data demonstrated that injured AECIIs were the main source of accumulated lipids in response to Bleomycin stimulation. Furthermore, we identified that lipids such as LysoPCs released by injured AECIIs could activate lung fibroblast, thus promoting pulmonary fibrosis progression in vivo. We further demonstrated that HMGCS2 played a vital role in mitigating lung fibrotic progression via modulating lipid degradation in AECIIs by promoting CPT1A and CPT2 expression by interacting with PPARα (Fig. 8).

In summary, our study not only unveiled a novel mechanism by which AECII-derived lipid molecules act as pro-fibrotic factors but also provided an appealing target for anti-lung fibrotic drug development.

### Supplementary Information

**Supplementary Material 1.**

**Supplementary Material 2.**

**Supplementary Material 3.**

**Supplementary Material 4.**

**Supplementary Material 5.**

**Supplementary Material 6.**

## Data Availability

All data generated or analyzed during this study are included in this published article; further inquiries can be directed to the corresponding author.

## Abbreviations

IPF: Idiopathic pulmonary fibrosis
AECIIs: Type II alveolar epithelial cells
BALF: Bronchoalveolar Lavage Fluid
KEEG: Kyoto Encyclopedia of Genes and Genomes
SASP: Senescence-associated secretory phenotype
FEV: Forced expiratory volume
FVC: Forced vital capacity
DLCO: Diffusing capacity for carbon monoxide
LysoPC: Lysophosphatidylcholine
PC: Phosphatidylcholine
PE: Phosphatidylethanolamine
PS: Phosphatidylserine
PG: Phosphatidylglycerol
Cer: Ceramide
SM: Sphingomyelin
