# Supplementary figures and images for: Downregulation of HMGCS2 mediated AECIIs lipid metabolic alteration promotes pulmonary fibrosis by activating fibroblasts
Source: Respir Res. 2024 Apr 24;25:176. doi: 10.1186/s12931-024-02816-z (PMC11040761; doi:10.1186/s12931-024-02816-z)

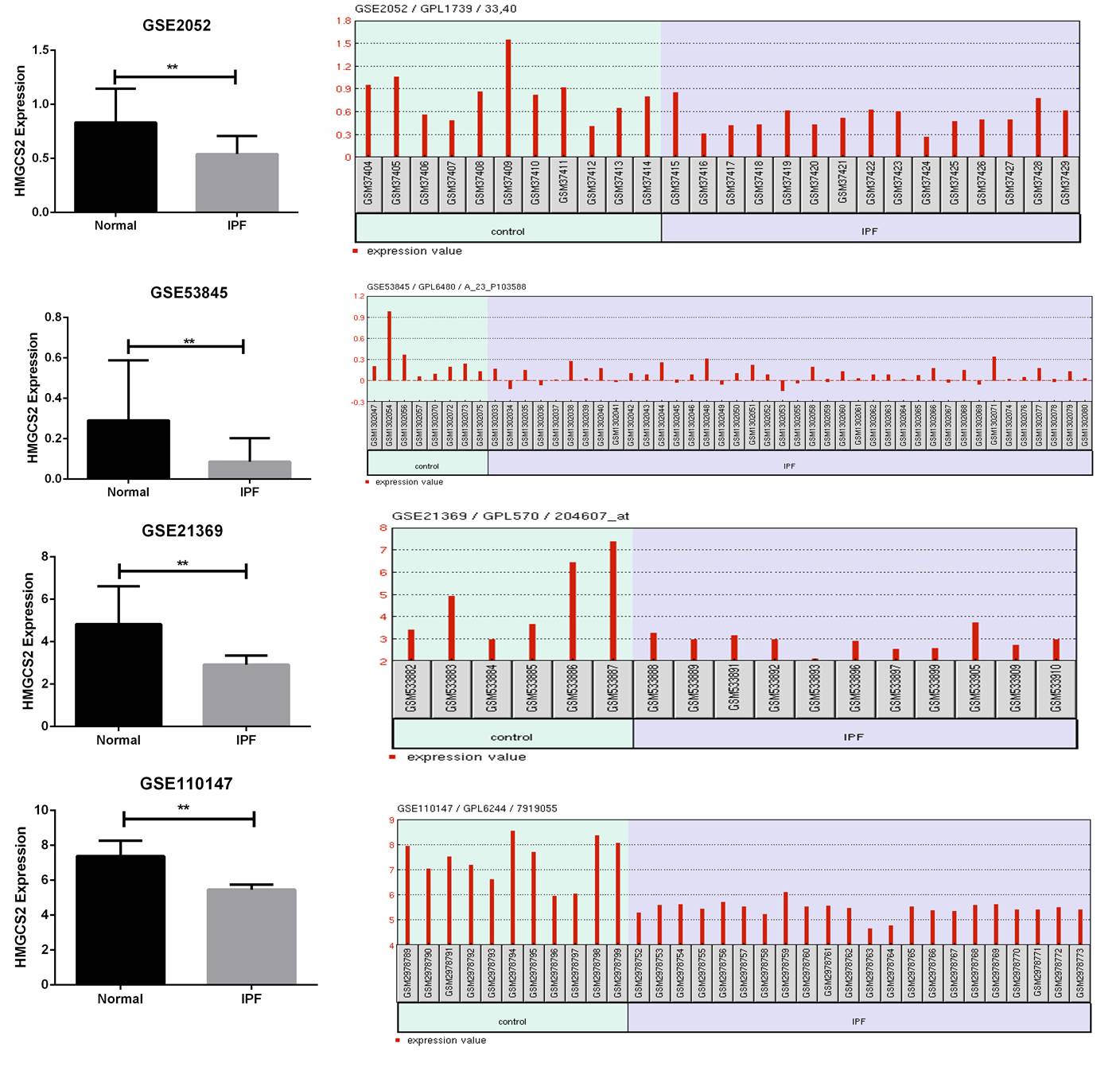

Supplement: Supplementary file 1 — Supplementary Material 1. [file 12931_2024_2816_MOESM1_ESM.jpg]

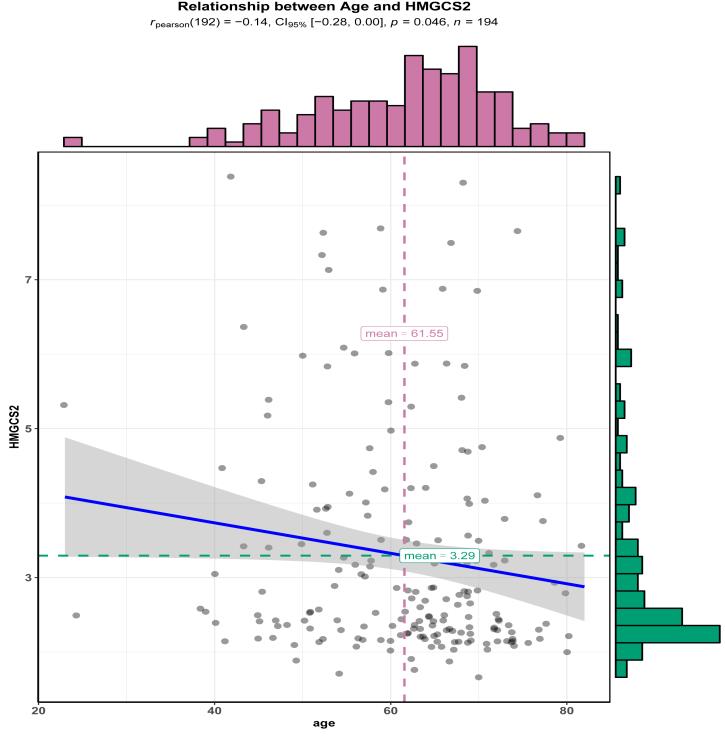

Supplement: Supplementary file 2 — Supplementary Material 2. [file 12931_2024_2816_MOESM2_ESM.jpg]

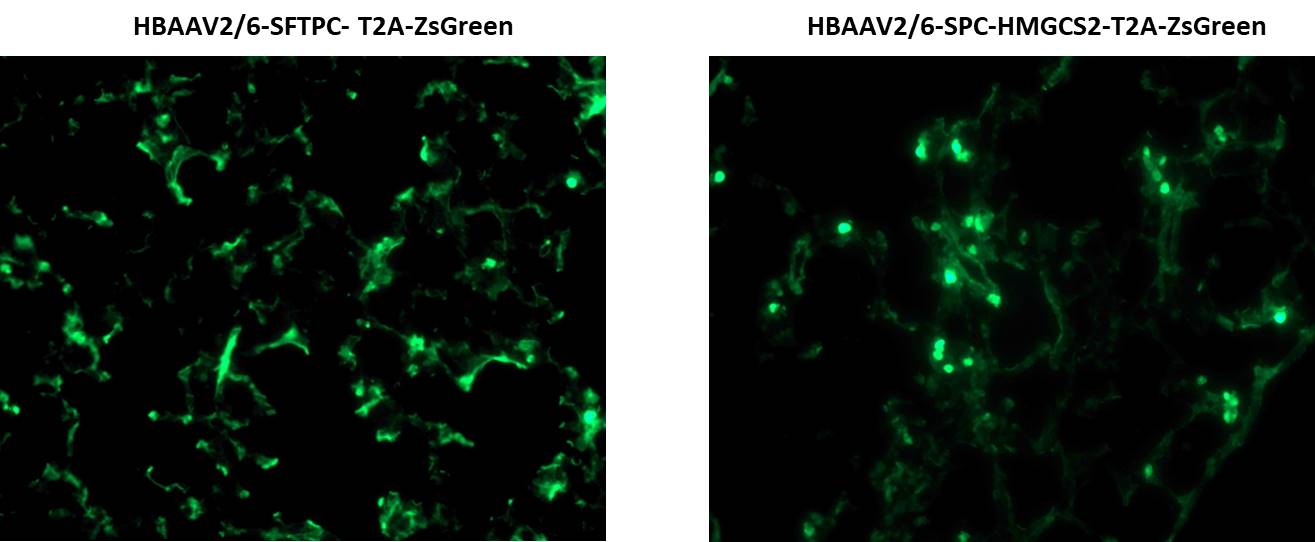

Supplement: Supplementary file 3 — Supplementary Material 3. [file 12931_2024_2816_MOESM3_ESM.jpg]

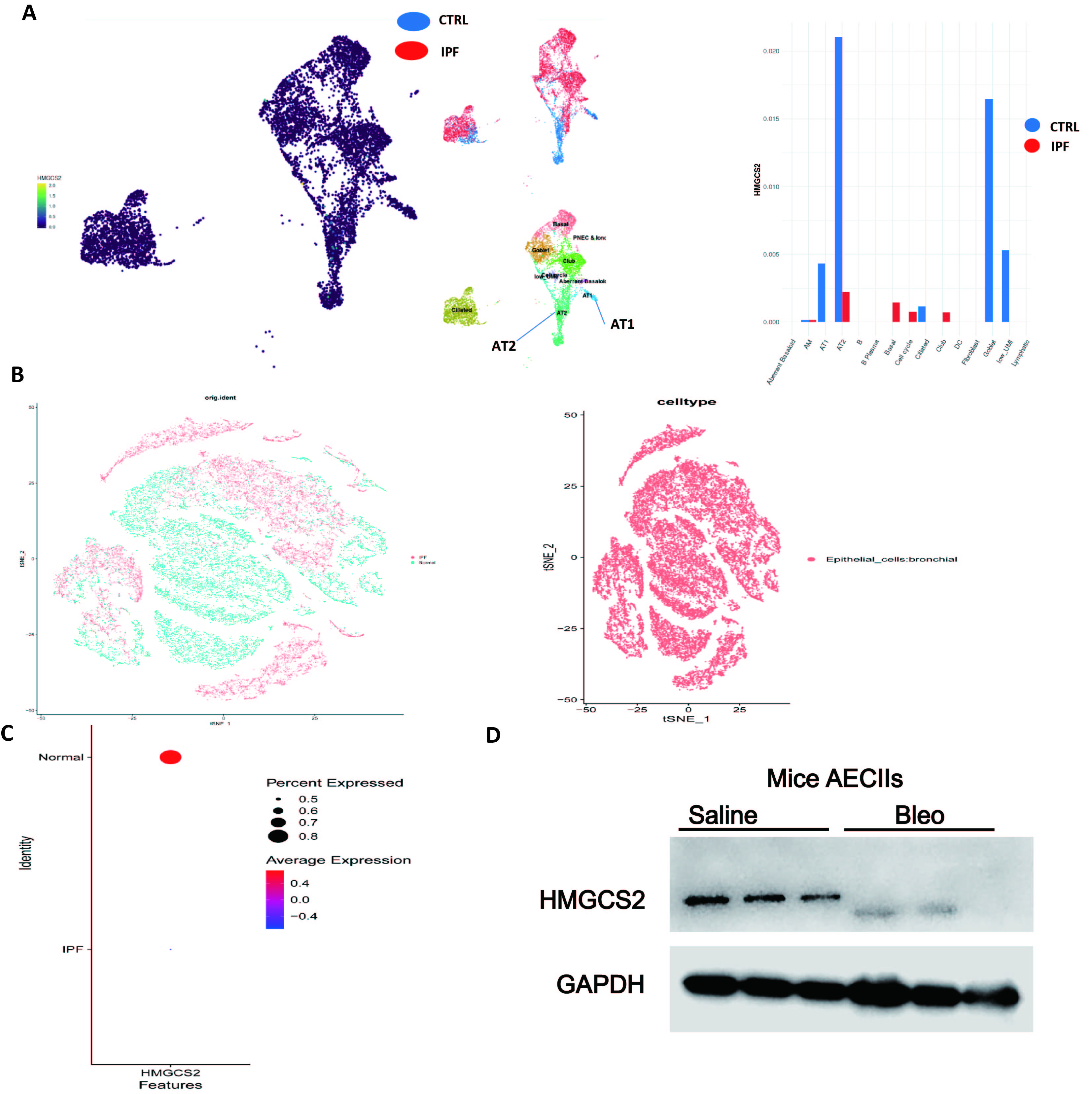

Supplement: Supplementary file 4 — Supplementary Material 4. [file 12931_2024_2816_MOESM4_ESM.jpg]
