# Supplementary material for: Downregulation of HMGCS2 mediated AECIIs lipid metabolic alteration promotes pulmonary fibrosis by activating fibroblasts
Source: Respir Res. 2024 Apr 24;25:176. doi: 10.1186/s12931-024-02816-z (PMC11040761; doi:10.1186/s12931-024-02816-z)
Supplement: Supplementary file 5 — Supplementary Material 5. [file 12931_2024_2816_MOESM5_ESM.docx]

Figure2D


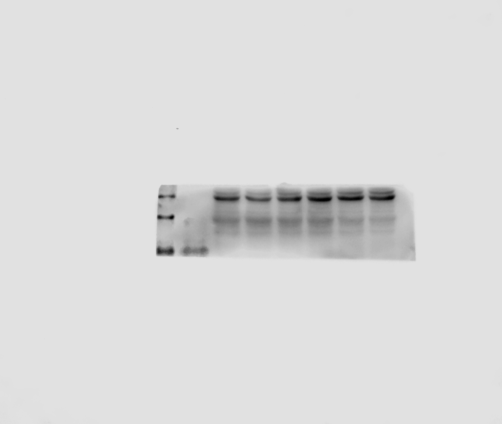


α-SMA


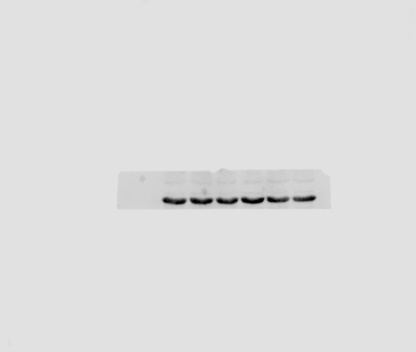


GAPDH

Figure2E


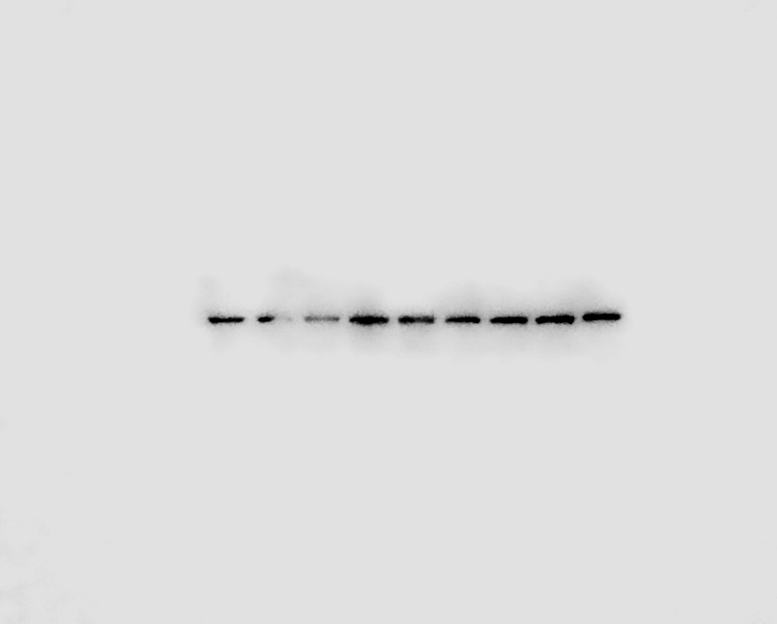


Collagan1

Fibronectin


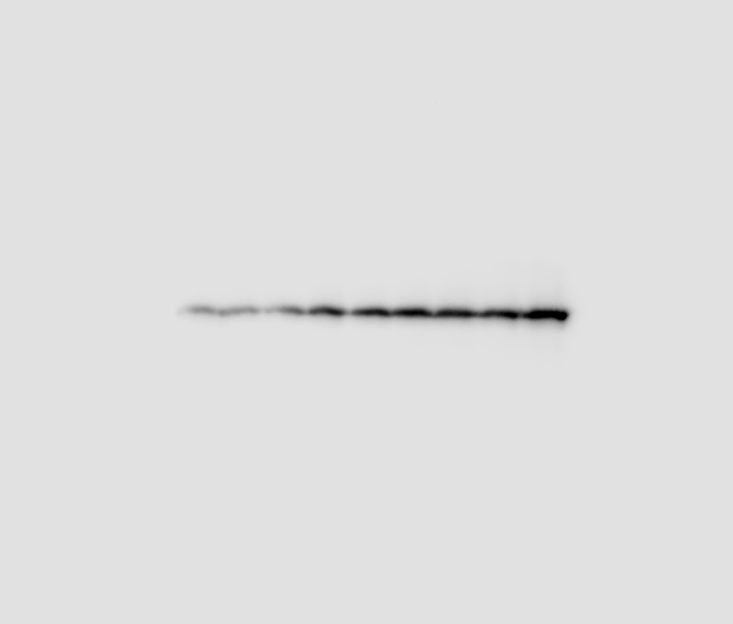


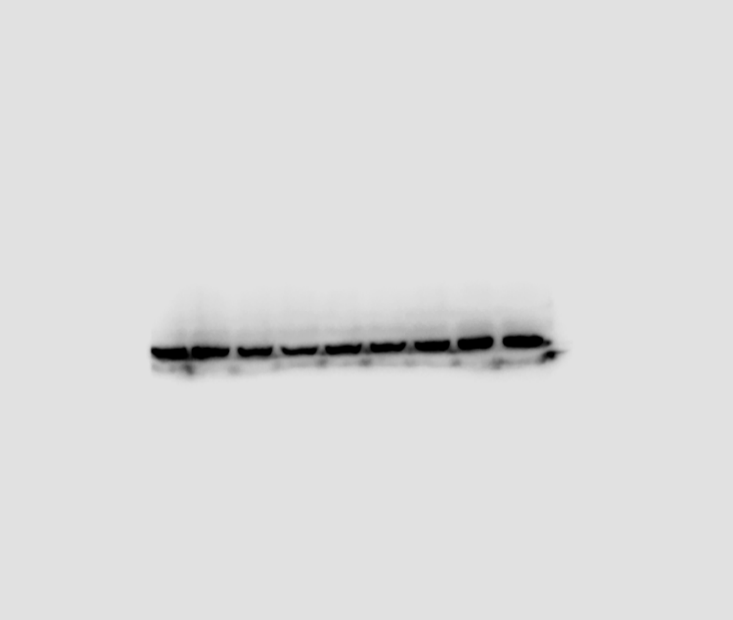


GAPDH

α-SMA

Figure2G


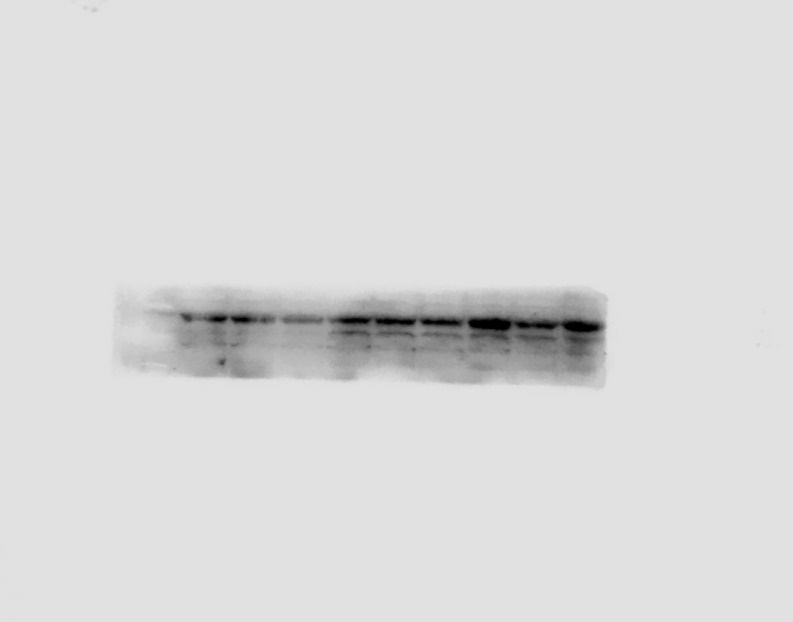


Fibronectin


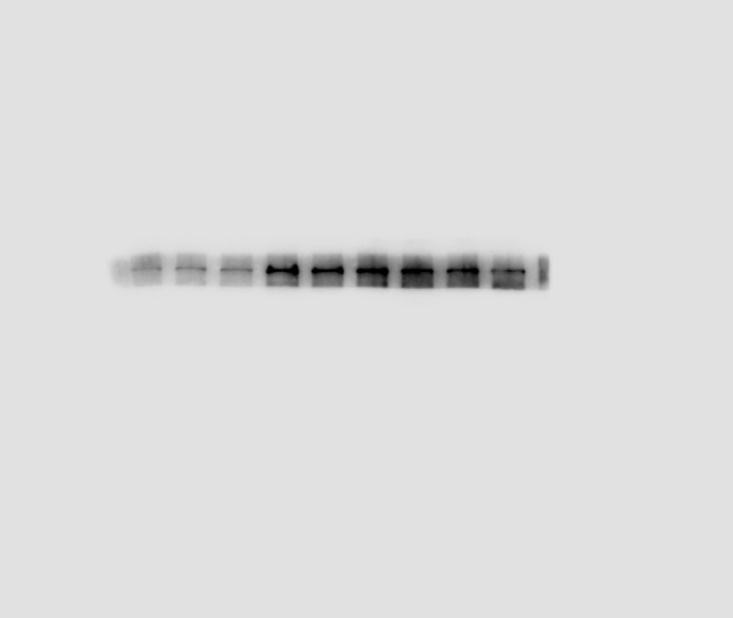


Collagan1


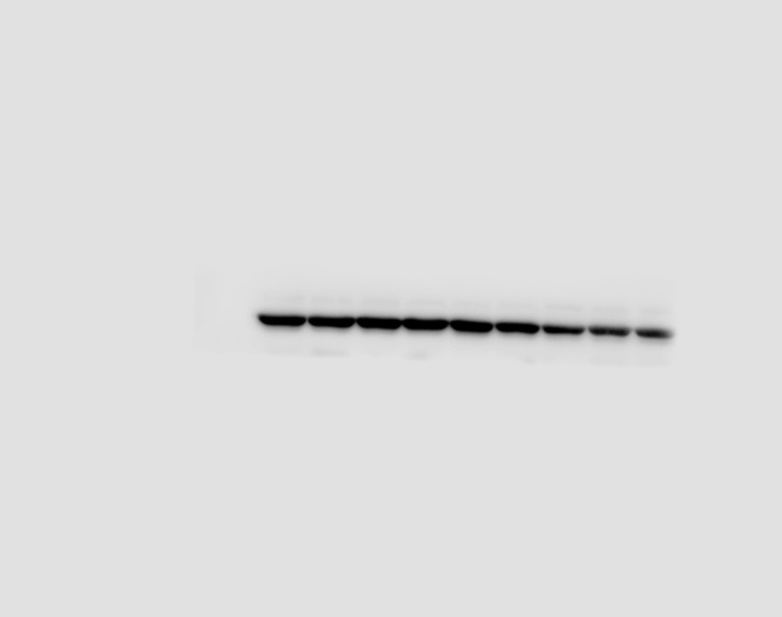


GAPDH

Figure4 B


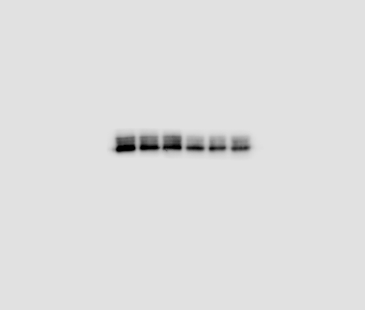


HMGCS2


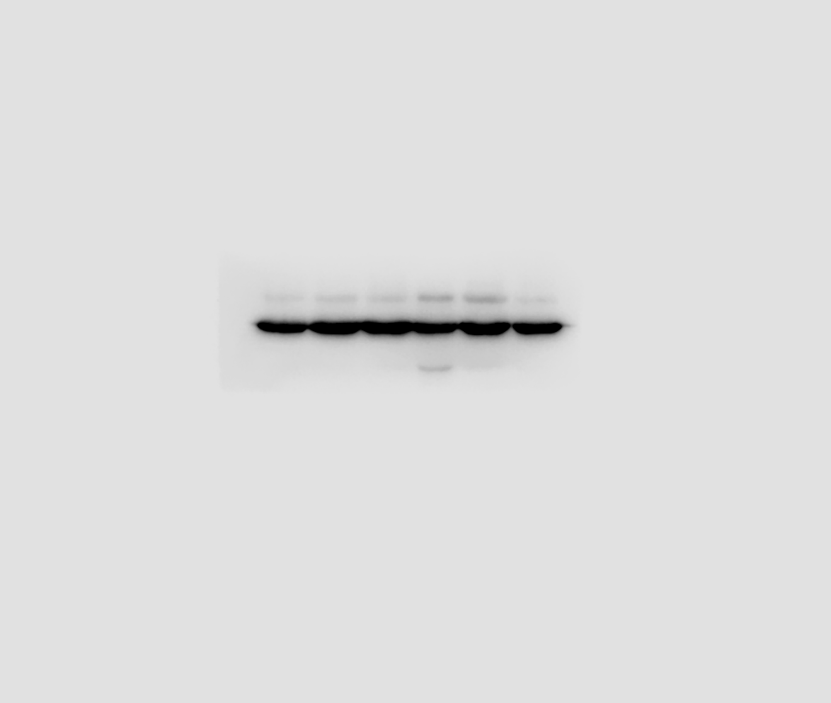


GAPDH

Figure 5A

Four separate A549 HMGCS2 stable expression single clones were determined

V1 H2-1 V2 H2-2 V3 H2-3 V4 H2-4


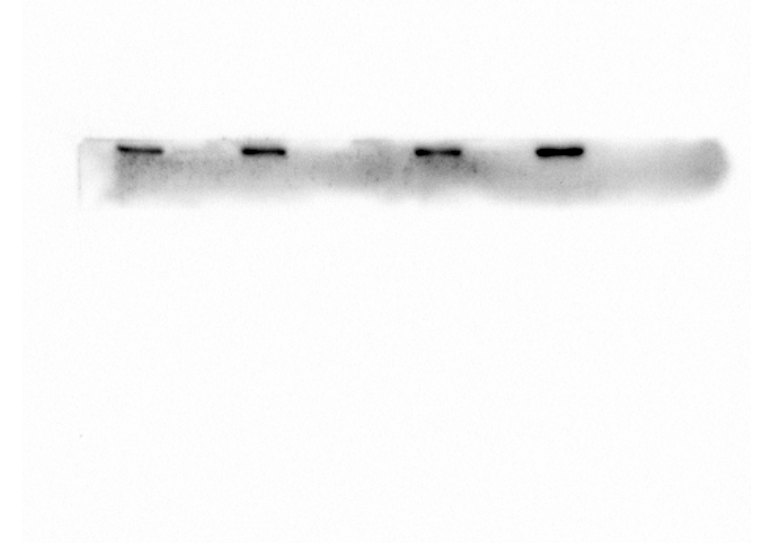

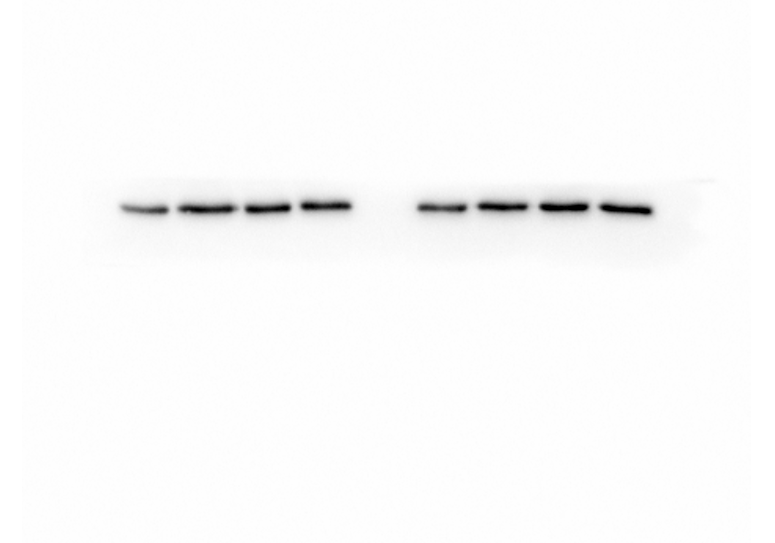


GAPDH

HMGCS2

Figure6D


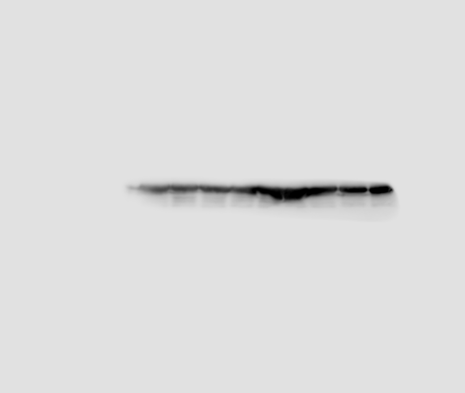


HMGCS2


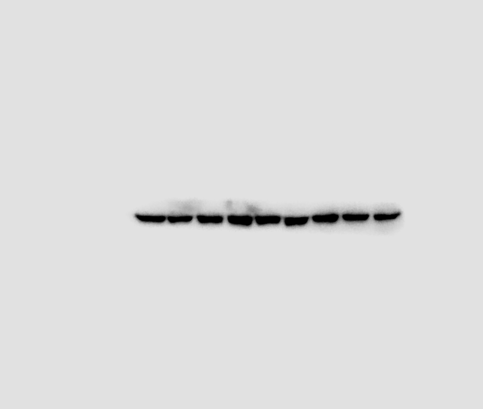


GAPDH


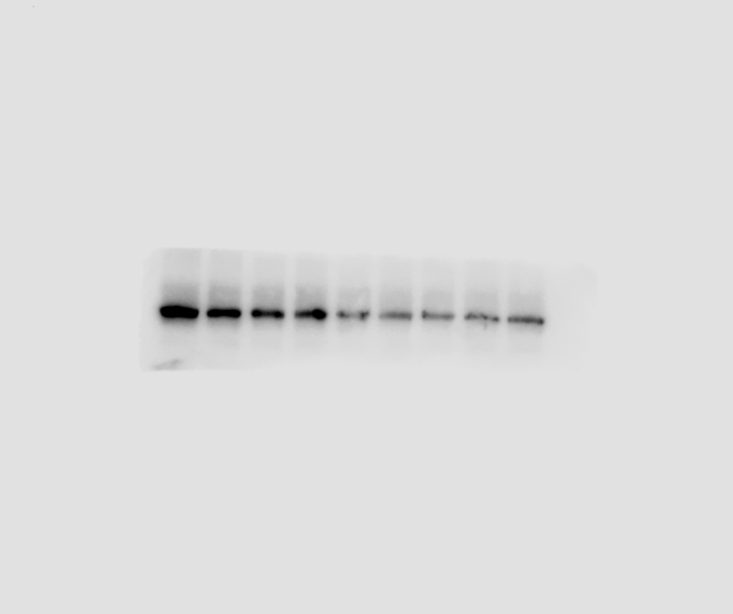


Collagan1


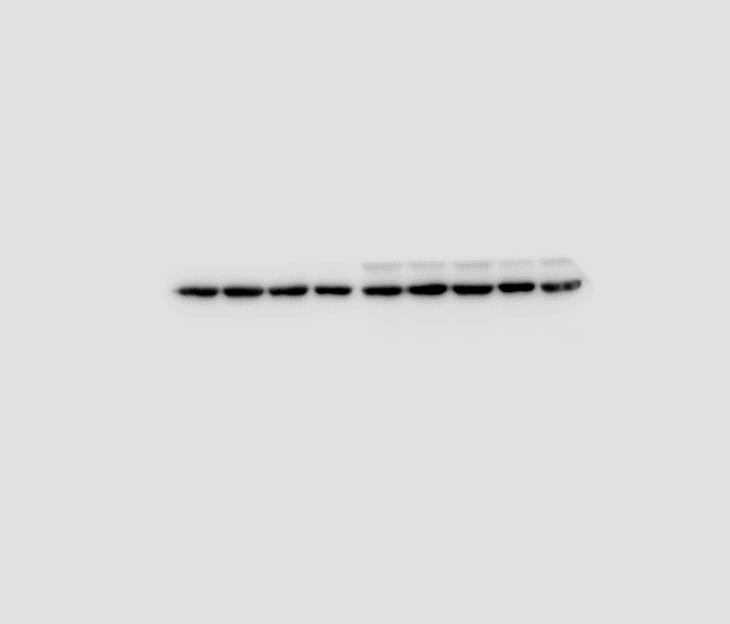


GAPDH

Figure 7B


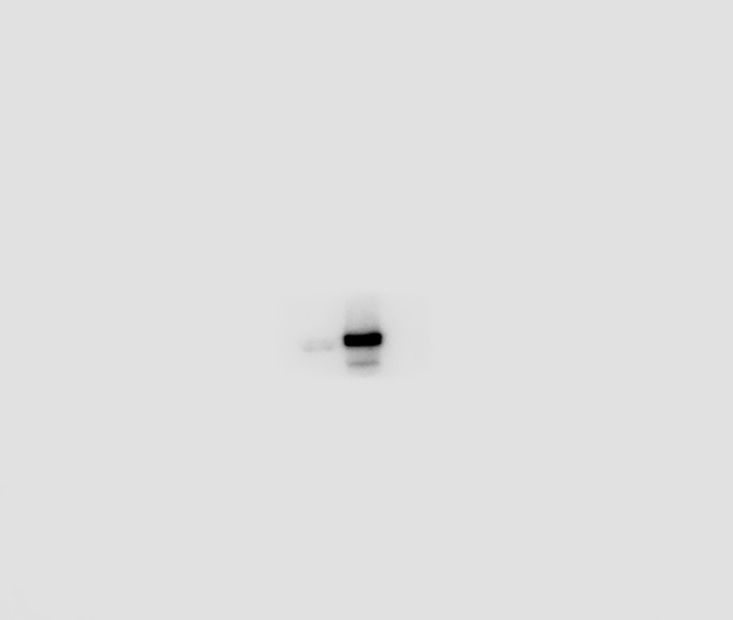


IP HMGCS2


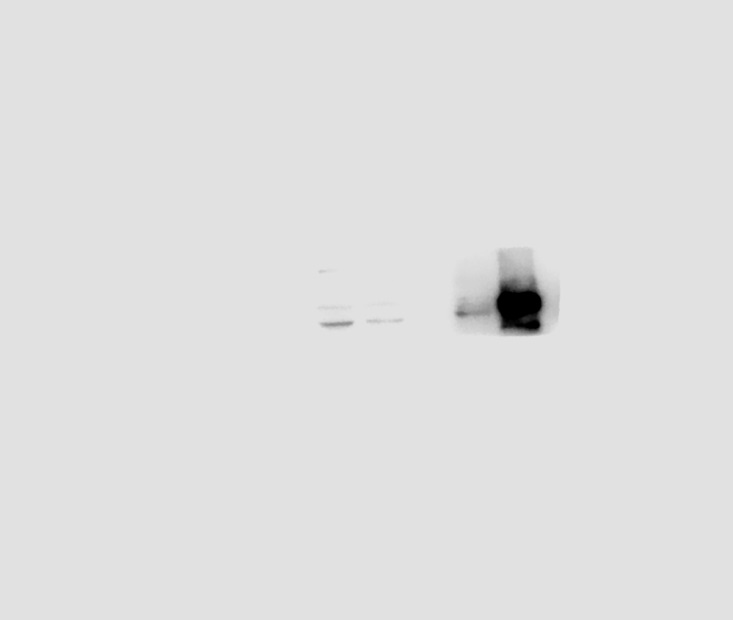


IP PPARα

Input PPARα


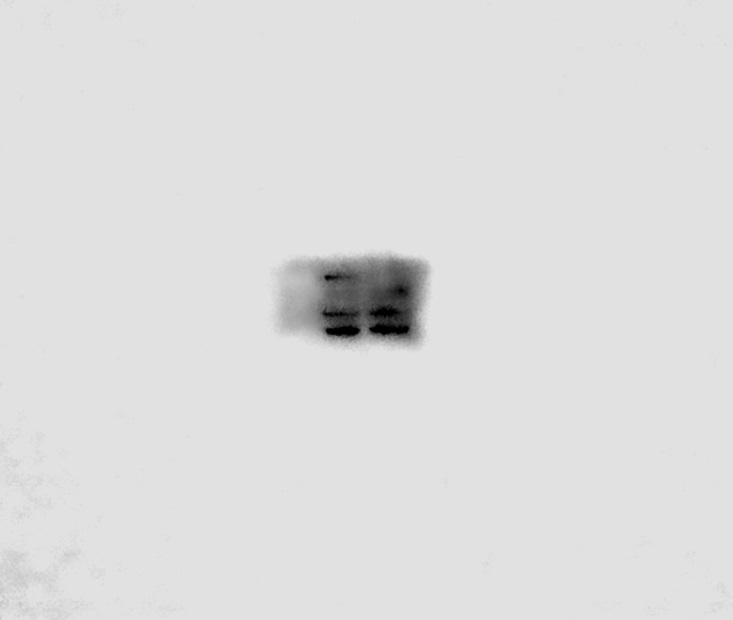


Input PPARα


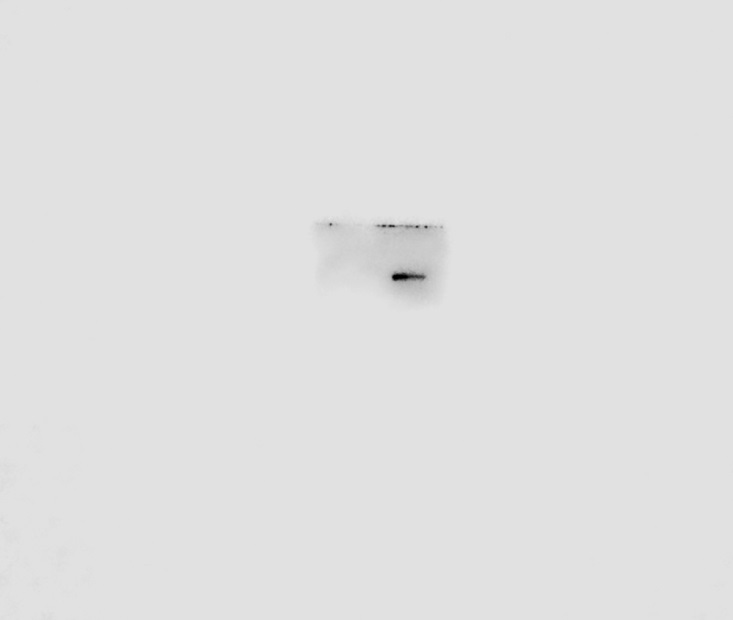


Input HMGCS2


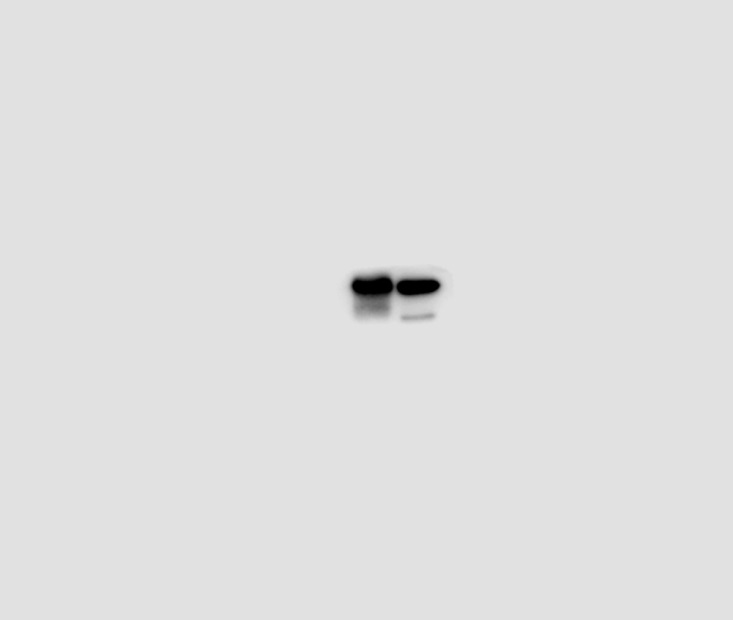


Input GAPDH

Figure7 C


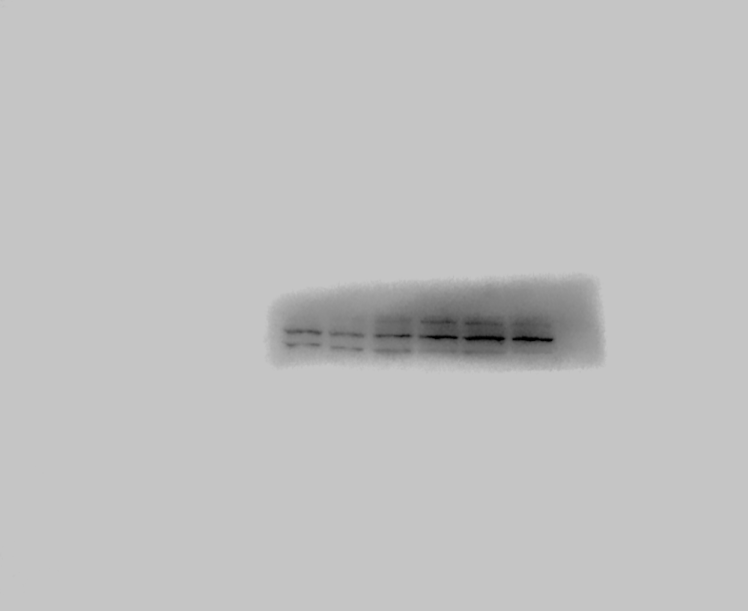


CPT2


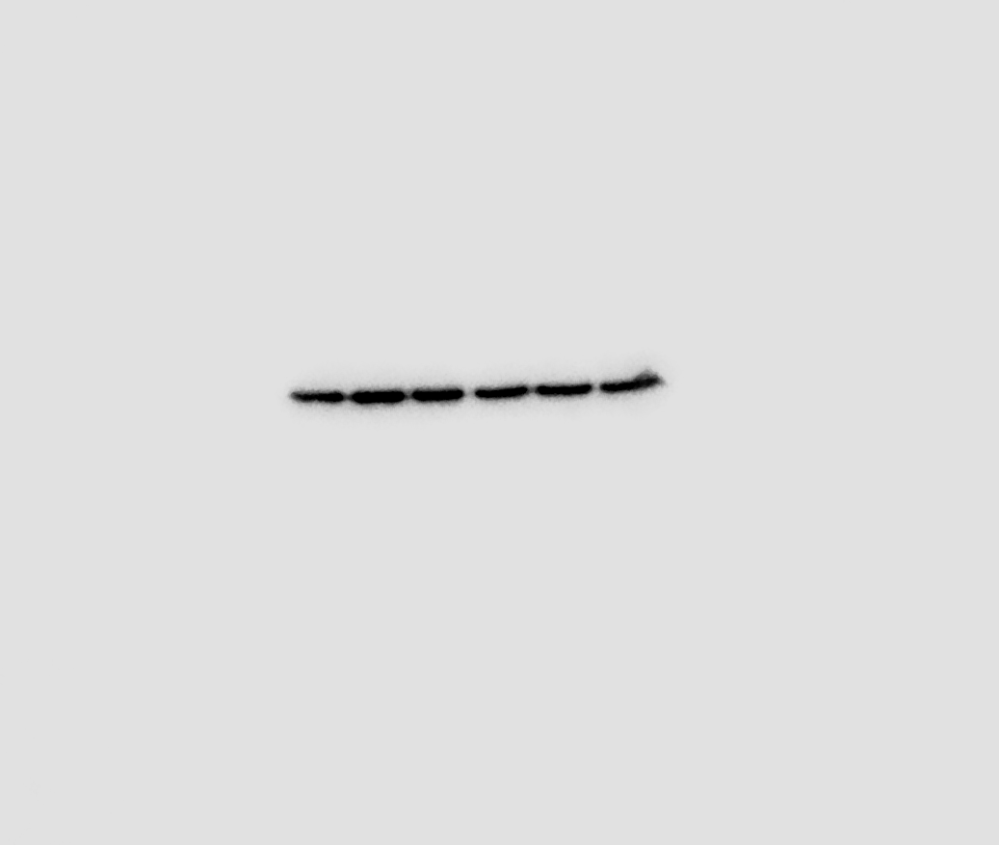


GAPDH


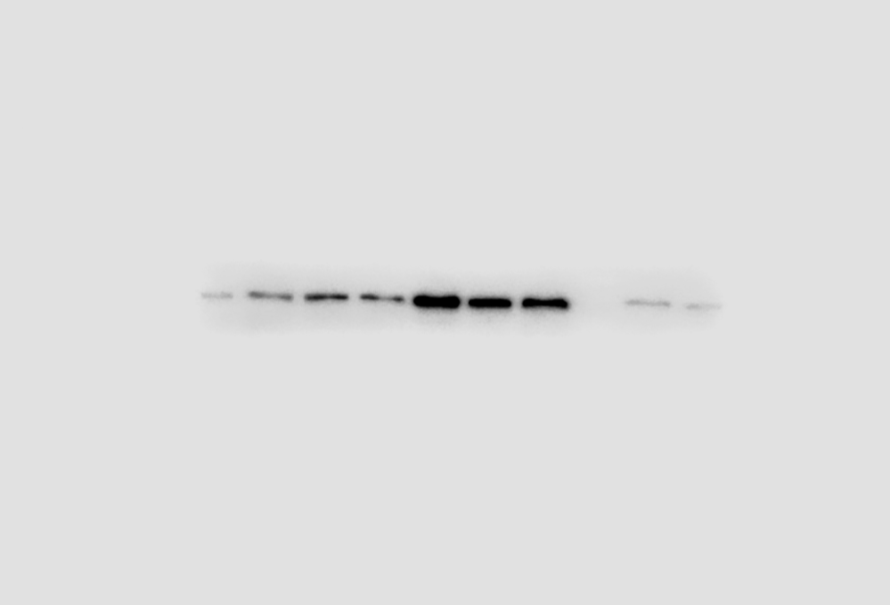


CPT1A


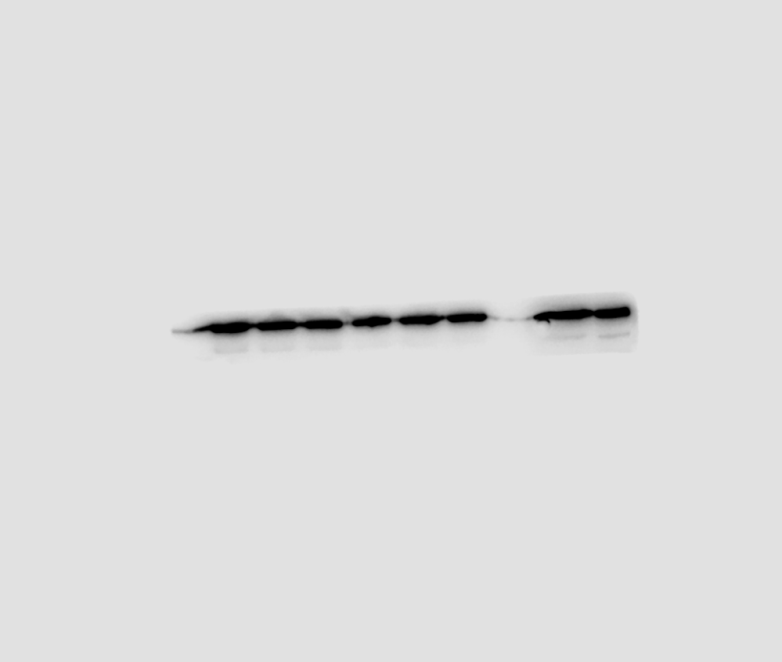


GAPDH

Figure 7D


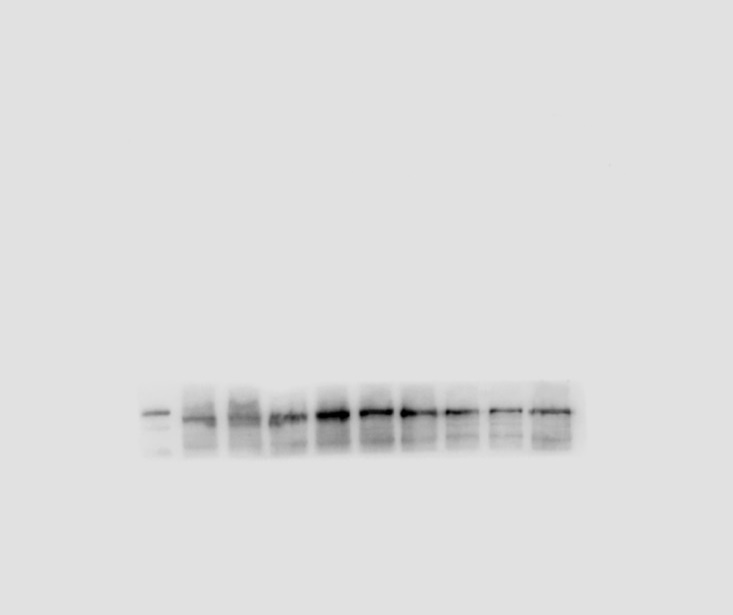


CPT1A


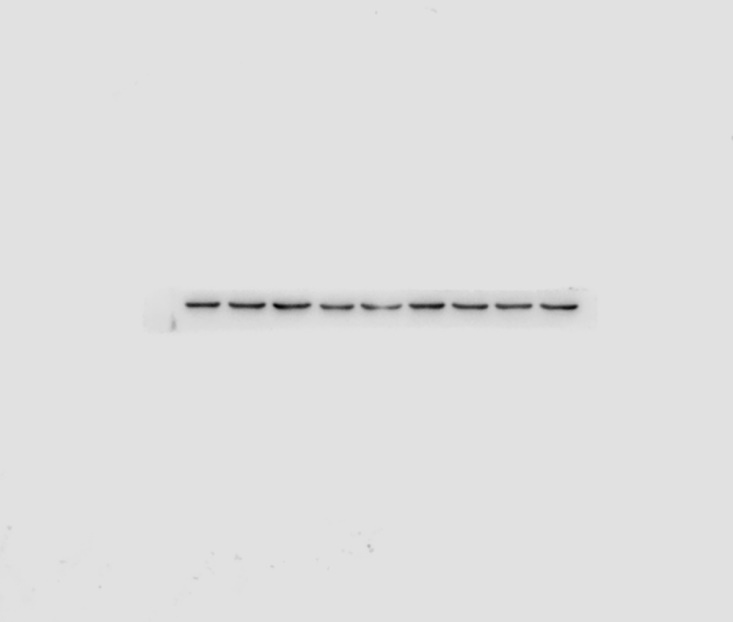


GAPDH


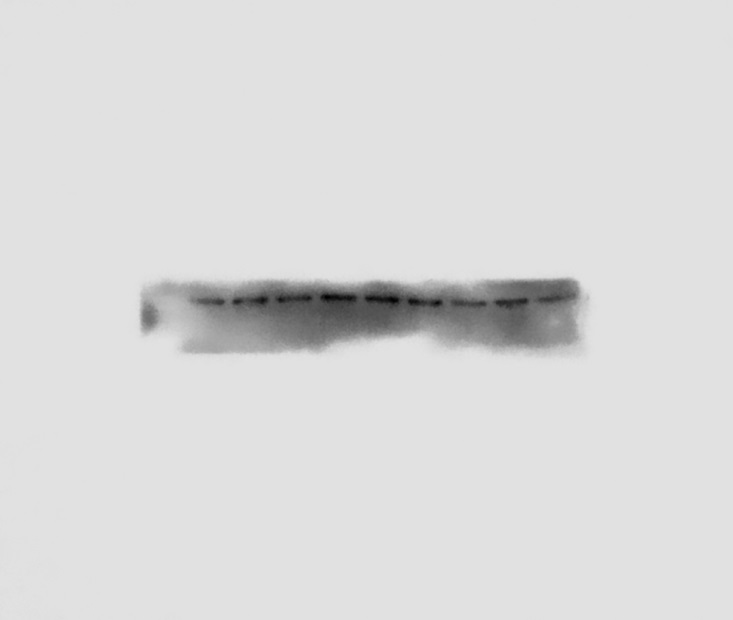


CPT2


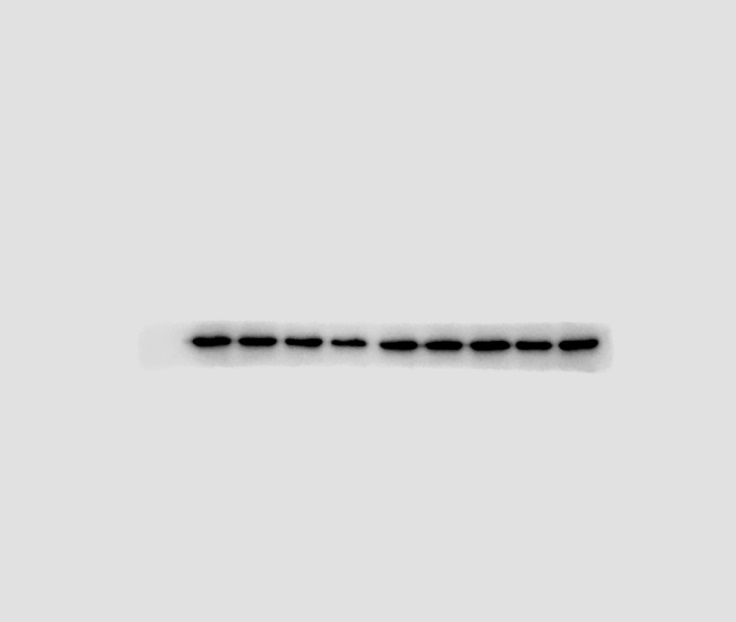


GAPDH

Figure S3 D


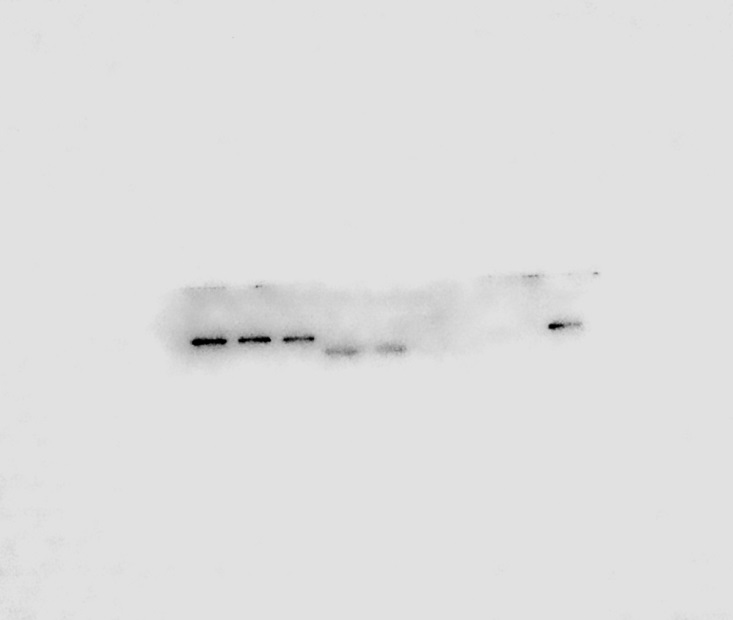

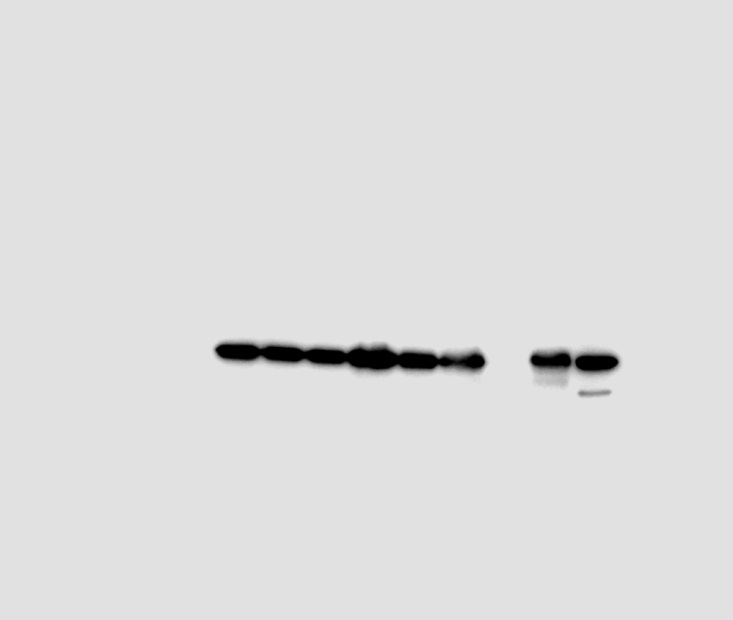


HMGCS2

GAPDH
