# Supplementary material for: Downregulation of HMGCS2 mediated AECIIs lipid metabolic alteration promotes pulmonary fibrosis by activating fibroblasts
Source: Respir Res. 2024 Apr 24;25:176. doi: 10.1186/s12931-024-02816-z (PMC11040761; doi:10.1186/s12931-024-02816-z)
Supplement: Supplementary file 6 — Supplementary Material 6. [file 12931_2024_2816_MOESM6_ESM.docx]

**Supplementary material**

**Figure S1.** The down regulation of HMGCS2 in IPF patients was further confirmed in other cohorts. HMGCS2 expression data were derived from GEO data base (GSE2052, GSE53845, GSE21369, GSE110147), results expressed as mean±SD. *: p<0.05; **: p<0.01;

**Figure S2** HMGCS2 expression was negatively associated with age in IPF patients

HMGCS2 expression and IPF patients’ information were derived from our previous used data (GSE47460- GPL14550)

**Figure S3** HMGCS2 was specifically down regulated in AECIIs

A, Data from IPF cell atlas ( <http://ipfcellatlas.com/>). B, IPF Single cells data was acquired from GEO (GSE132915) followed by standard data analysis pipeline, the AECII cells were identified by SFTPC. C, HMGCS2 expression was significantly down regulated in AECIIs. D, AECIIs were isolated in saline and Bleomycin treated mice and HMGCS2 was significantly decreased upon Bleomycin injury.

**Figure S4.** Evaluation of adeno-associated virus infection efficiency

The frozen lung section of HBAAV2/6-SFTPC- T2A-ZsGreen and HBAAV2/6-SPC-HMGCS2-T2A-ZsGreen infected mice was examined using fluorescence microscope 3 weeks after infection.

Table S1 Primers used in this study

| Primer name | Oligonucleotide sequence (5’-3’) Usage |
| --- | --- |
| L393V-F  L393V-R | 5'-TGCCTGGCCTCGGTTCTGTCCCACC-3' Pointmutation 5'-GGTGGGACAGAACCGAGGCCAGGCA-3 Point mutation |

Table S2 LysoPC with compound ID identified by lipid omics

| **Lipid Class** | **Compound ID** | **Fold Change** |
| --- | --- | --- |
| LysoPC(14:0) | HMDB0010379 | 2.55597 |
| LysoPC(15:0) | HMDB0010381 | 2.66085 |
| LysoPC(16:0) | HMDB0240262 | 2.30832 |
| LysoPC(18:3) | HMDB0010388 | 0.33127 |
| LysoPC(20:1) | HMDB0010391 | 2.16471 |
| LysoPC(20:4) | HMDB0010396 | 2.00343 |
| LysoPC(22:6) | HMDB0010404 | 2.12753 |
